# Macrodrop‐Impact‐Mediated Fluid Microdispensing

**DOI:** 10.1002/advs.202101331

**Published:** 2021-06-26

**Authors:** Shiji Lin, Dehui Wang, Lijuan Zhang, Yakang Jin, Zhigang Li, Elmar Bonaccurso, Zili You, Xu Deng, Longquan Chen

**Affiliations:** ^1^ School of Physics University of Electronic Science and Technology of China Chengdu Sichuan 611731 P. R. China; ^2^ Institute of Fundamental and Frontier Sciences University of Electronic Science and Technology of China Chengdu Sichuan 610054 P. R. China; ^3^ School of Life Science and Technology Center for Informational Biology University of Electronic Science and Technology of China Chengdu Sichuan 610054 P. R. China; ^4^ Department of Mechanical and Aerospace Engineering The Hong Kong University of Science and Technology Clear Water Bay Kowloon Hong Kong SAR P. R. China; ^5^ AIRBUS Central R & T Materials X Munich 81663 Germany

**Keywords:** drop impact, fluid dispensing, jet dynamics, non‐wetting surfaces

## Abstract

High‐resolution fluid dispensing techniques play a critical role in modern digital microfluidics, micro‐biosensing, and advanced fabrication. Though most of existing dispensers can achieve precise and high‐throughput fluid dispensing, they suffer from some inherent problems, such as specially fabricated dispensing micronozzles/microtips, large operating systems, low volume tunability, and poor performance for low surface tension liquids and liquids containing solid/liquid additives. Herein, the authors propose a facile, low‐frequency micro dispensing technique based on the Rayleigh–Plateau instability of singular liquid jets, which are stimulated by the air cavity collapse arising in the impact of microliter drops on non‐wetting surfaces. This novel dispensing strategy is capable to produce single microdrops of low‐viscosity liquids with a tunable volume from picoliters to nanoliters, and the operational surface tension range covers most laboratory solvents. The dispensing function is implemented without using small‐dimension nozzles/tips and enables handling diverse complex liquids. Moreover, the rather simple operating platform allows the integration of the whole dispensing function into a handy portable device with a low cost. Employing this microdispensing technique, the authors have controlled microchemical reactions, handled liquid samples in biological analysis, and fabricated smart materials and devices. The authors envision that this rational microdrop generator would find applications in various research areas.

## Graphical abstract

Being free from using small‐dimension tips/nozzles or large operating platforms remains a big challenge in existing dispensing techniques with high volume tunability and liquid compatibility. Based on the jet dynamics arising in macrodrop impact on non‐wetting surfaces, a novel microdispensing technique, which can produce single drops in the picoliter to nanoliter volume range for diverse simple and complex liquids, is proposed.

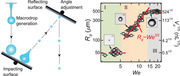

## 1 Introduction

Fluid dispensers with precise volume control, such as micropipettes^[^ ^1^ ^]^ and ink‐jets,^[^ ^2^, ^3^ ^]^ have been widely used for transferring small quantities of liquids in fundamental scientific research and in industrial processes. More specifically, fluid dispensing systems capable of delivering liquid volumes of a few microliters or less are of primary importance for prototyping of functional nanomaterials,^[^ ^4^, ^5^ ^]^ microchemical analysis,^[^ ^6^, ^7^ ^]^ and quantitative monitoring of gene expression or sequencing,^[^ ^8^, ^9^ ^]^ where physical, chemical, biological, or coupled processes are monitored in sessile drops. While the continuing miniaturization of those operating systems significantly improves working efficiency and reduces costs,^[^ ^7^, ^10^ ^]^ it also poses challenges to the size tunability and liquid compatibility of fluid dispensers, which are key for handling fluids with diverse properties on multifarious substrates in practical applications.

Dispensing techniques have been broadly categorized as contact and non‐contact.^[^ ^11^ ^]^ Contact dispensing utilizes diverse tips or nozzles to pick‐up, transfer, and deposit liquids by directly touching the target substrates. Though single‐drop volumes down to the attoliter range can be dispensed using sharp atomic force microscope tips,^[^ ^12^, ^13^, ^14^ ^]^ the application of this technique suffers from the complex microfabrication, the slow dispensing speed, and the fragility of dispensing tips/nozzles due to hard contact. Alternatively, pico‐ to nanoliter volume drops have been deposited on solid surfaces by the selective dip‐coating of dispensing liquids on chemically micropatterned surfaces,^[^ ^15^, ^16^ ^]^ but tuning the drop size involves a new round microfabrication in cleanrooms. By contrast, non‐contact dispensing allows on‐demand and high‐throughput liquid transfer with no risk of damaging the nozzle.^[^ ^17^ ^]^ Among various dispensing configurations, the simplest and most common one is to pump liquids through a drip nozzle, at which microliter drops form and fall when the gravity counterbalances the capillary force.^[^ ^17^, ^18^ ^]^ Further reduction in dispensing volume requires a scaled‐down nozzle size—which in turn generates high wall friction.^[^ ^19^ ^]^ As a solution, piezoelectric actuators or resistive heaters are employed to expel hydrodynamic jets from small nozzles, and thereby form picoliter drop series via the jet instability (i.e., the so‐called ink‐jet printing).^[^ ^20^, ^21^ ^]^ Such small drops can also be produced from laser‐induced jet emission,^[^ ^22^, ^23^ ^]^ electrohydrodynamic jetting,^[^ ^24^, ^25^ ^]^ pyroelectrodynamic shooting,^[^ ^26^ ^]^ acoustophoretic printing,^[^ ^27^ ^]^ and recently drop printing based on microfluidics.^[^ ^28^ ^]^ However, these dispensing methods—particularly the jet‐forming ones like the ink‐jetting system—rely on specially designed and fabricated small dispensing nozzles (Figure S1, Supporting Information), while others may have some specific requirements on the electromagnetic properties of dispensed liquids and target substrates (Table S1, Supporting Information). Moreover, all aforementioned techniques need rather large control platforms and do not allow to tune the drop volumes on demand (Figure S1, Supporting Information). Most of them also hardly dispense single drops of low surface tension liquids (e.g., surface tension should normally be higher than ≈ 35 mN m^−1^ for ink‐jet printing^[^ ^3^ ^]^ and acoustic injection^[^ ^29^ ^]^) and complex liquids containing surfactants or colloids, which would disrupt the jet stream or cause tip/nozzle contaminations and clogging.

In this work, we report a novel strategy for non‐contact fluid dispensing at a low frequency by exploiting the jet dynamics of impinging microliter drops on sufficiently lyophobic surfaces, which produces single drops with volumes ranging from picoliters to nanoliters. The jet emission and thus jet drop formation are the consequence of the collapse of the air cavity that forms through the development, propagation and oscillation of a capillary wave during drop impact. The dispensing function is implemented without complex control platforms, making it possible to integrate the entire operating system into a handy portable device. The use of common commercially available dispensing nozzles allows handling and manipulating not only pure low‐viscosity liquids but also surfactant‐laden liquids, colloidal suspensions, emulsions and biological fluids with surface tensions down to ≈ 25 mN m^−1^ and up to ≈ 72 mN m^−1^, covering most solvents for lab used. We believe that the proposed microdispensing technique would have broad applications in both scientific and engineering fields.

## 2 Results and Discussion

### 2.1 Jet Drop Formation in Macrodrop Impact

Jet emission is an intriguing outcome of the impact of low‐viscosity drops on non‐wetting surfaces.^[^ ^30^, ^31^ ^]^ We can readily show such jetting by impinging microliter water drops (radius *R*_0_ = 1.0 − 2.0 mm) onto superhydrophobic surfaces at a velocity (*V*_0_) of 0.4 − 1.5 m s^‐1^. The corresponding Ohnesorge number $h\;=\;\mu /\sqrt{\rho R_{0}\gamma }$, which compares the viscous force to the inertial and surface tension forces, is typically on the order of 10^−3^, suggesting that the impact processes would be dominated by the inertial and surface tension forces and thus the Weber number $We=\rho R_{0}V_{0}^{2}/\gamma$ (3≲We≲30) can be employed to characterize the impact dynamics, where µ, *ρ*, and *γ* denote the liquid viscosity, density, and surface tension, respectively. The superhydrophobic surfaces were fabricated by coating silicon substrates with a thin layer of silica nanospheres (Figure S2, Supporting Information) and further perfluoro‐silanized to achieve excellent liquid repellency.^[^ ^32^ ^]^ The apparent contact angle and contact angle hysteresis for 4 µL water drops were measured to be *θ*_app_ = 160 ± 1° and $\Delta \theta =4\pm 2^{\circ }$. The drop dynamics was viewed and monitored from the side using a high‐speed camera with a recording rate of up to 340 000 fps.

**Figure 1:**
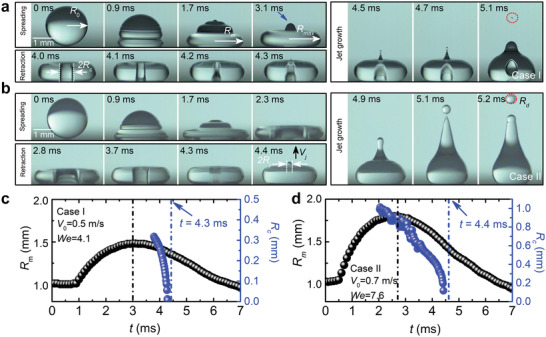
a,b) Time‐lapsed images of impinging water drops (*R*_0_ ≈ 1.0 mm) on a superhydrophobic surface at Weber numbers *We* ≈ 4.1 (Case I) and *We* ≈ 7.6 (Case II), respectively. c,d) Temporal evolution of the maximum curvature radius (*R_m_*) of the impinging drop and the cavity radius (*R_c_*) for impact events in Case I (a) and Case II (b). The black and blue dashed lines denote the times of maximum drop spreading and cavity collapse, which divide the drop impact process into three sequential parts: spreading, retraction, and jet emission as indicated in (a) and (b).

An impinging drop usually undergoes spreading, retraction, and jet emission on a non‐wetting surface,^[^ ^30^, ^33^ ^]^ and we have observed two distinct jetting scenarios. **Figure** **1a** and Movie S1, Supporting Information, present a representative phenomenon of drop impact at We≲6. Upon hitting the surface, the instantaneous compression of the liquid generates a capillary wave that propagates from the bottom to the top of the impinging drop,^[^ ^34^ ^]^ shaping the drop into a pancake structure with a spire (denoted by the blue arrow at 3.1 ms in Figure 1a) at the maximum spreading. The further downward oscillation of the spire results in a cylindrical air cavity at the drop center, which subsequently collapses axially inward with the recoiling drop (see the evolution of the drop radius *R_m_* and the cavity radius *R_c_* in Figure 1c). The cavity keeps its cylindrical shape at the beginning, and then gradually evolves into a flask‐like structure since its top retraction becomes sufficiently fast (4.0 − 4.3 ms in Figure 1a). As the top of the cavity closes, a sub‐millimeter‐sized bubble is entrapped and shortly afterward (within ≈ 17 µs) a thin jet shoots out (4.3 − 4.7 ms in Figure 1a). In comparison, at We>∼6, the fast drop spreading causes the spire and thus the air cavity to form earlier (Figure 1b and Movie S2, Supporting Information). As such, cavity collapse starts prior to the drop recoiling (Figure 1d), and consequently a thick jet is ejected. These jet emissions only occur for impinging low‐viscosity drops (μ≲10mPa·s) on sufficiently hydrophobic surfaces (θapp>∼108∘), and are particularly robust on superhydrophobic surfaces, regardless of their microscopic surface structures (Figure S3, Supporting Information).

To investigate the jetting phenomena in detail, we used impact events of water drops on the nanostructured superhydrophobic surface in Figure 1a,b. We found that both the jet radius (*R_j_*) and the jet speed (*V_j_*) exhibit a non‐monotonic dependence on the impact velocity, consistent with previous studies.^[^ ^31^ ^]^ The jetting characteristics can be divided into three distinct regimes (Figure S4a, Supporting Information): At low Weber numbers (3≲We≲6, regime I), *R_j_* is typically 20 − 35 µm while *V_j_* ranges from 2.3 to 8.2 m s^−1^; at intermediate Weber numbers (6≲We≲15, regime II), *R_j_* increases from ≈ 76 to ≈ 296 µm while *V_j_* decreases from ≈ 2.8 m s^−1^ to ≈ 1.8 m s^−1^; at high Weber numbers (15≲We≲20, regime III), *R_j_* approaches an asymptotic value of ≈ 300 µm and *V_j_* keeps nearly constant at ≈ 2.0 m s^−1^. By re‐plotting the data in the form of *V_j_* versus *R_j_* (Figure S4b, Supporting Information), power‐law correlations, $V_{j}\propto R_{j}^{-\alpha }$, are identified. For thin jets ($R_{j}≲35\;\mu m$) produced through the axial implosion of the air cavity, the obtained exponent *α* is close to 1, in good agreement with the theoretical prediction based on the conservation of mass and energy.^[^ ^31^ ^]^ On the other hand, the formation of thick jets (Rj>∼76μm) bears some similarity with the jet emission in bubble bursting,^[^ ^35^, ^36^ ^]^ which originates from the focusing of the capillary wave. As a result, the data points fall approximately on the scaling $V_{j}\propto R_{j}^{-1/2}$, as suggested by dimensional analysis (Text S1, Supporting Information).

**Figure 2:**
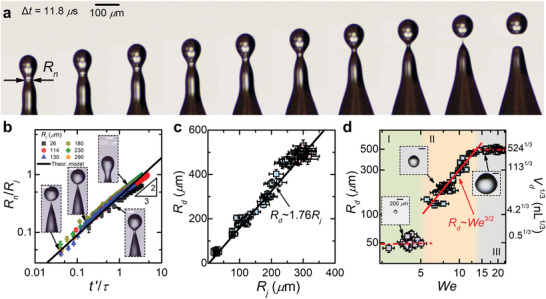
a) Snapshots of the pinch‐off dynamics on a singular jet with radius *R_j_* ≈ 30 µm. b) Normalized neck radius *R_n_*/*R_j_* as a function of the normalized time *t*′/*τ*. The black line corresponds to the theoretical prediction. c) Drop radius *R_d_* versus jet radius *R_j_*. The solid line is the linear fit of the experimental data, *R_d_* ≈ 1.76*R_j_*. d) Jet drop radius *R_d_* and volume *V_d_* versus the Weber number *We* in log‐log coordinates for impinging macrodrops with radii *R*_0_ ≈ 1.0 mm. The green‐, orange‐, and gray‐shaded regions correspond to three jetting characteristics regimes.

In the further rising, Rayleigh–Plateau instability is stimulated spontaneously, as evidenced by the propagating waves and the formation of bulges on the jet. Ultra‐high speed imaging reveals that the neck bridging the top two bulges rapidly thins into a singular point and eventually breaks up under the action of surface tension within a timescale of $\tau ={\left(\rho R_{j}^{3}/\gamma \right)}^{1/2}$ (≈ 20 µs for the jet with *R_j_* ≈ 30 µm in **Figure** **2a**), generating a small drop flying off in the same direction and at the same velocity as the jet (Figure S5 and Movies S3 and S4, Supporting Information). Note that the jet instability at We>∼20 can produce two or three small drops (Figure S3, Supporting Information), and we only analyzed the dynamics of the first drop thereafter. The liquid thinning process, which is characterized by the evolution of the minimum neck radius *R_n_*, obeys a *t*′^2/3^ scaling (Figure 2b), with *t*′ being the relative time to breakup. The collapse of the nondimensionalized data for different jet sizes using the initial jet radius (*R_j_*) and the capillary‐inertial time (*τ*) in Figure 2b also suggests that the pinch‐off is inertia dominated and proceeds with a self‐similar behavior.^[^ ^20^, ^37^ ^]^ Moreover, the thinning neck induces a contraction flow along the injection axis,^[^ ^38^ ^]^ increasing the size of the top bulge until it pinches off. The volume of the jet drop can thus be estimated as

(1) $$V_{d}\approx \int_{0}^{\tau_{p}}\pi R_{n}^{2}\bar{V}dt^{\prime }+2\pi R_{j}^{3}$$

where $R_{n}\approx 0.42R_{j}{\left(\frac{t^{\prime }}{\tau }\right)}^{2/3}$is obtained from the power‐law fitting; *τ*_*p*_ ≈ 3.7*τ* is the duration of the pinch‐off process; $\bar{V}$ is the average velocity of the contraction flow, which is of the order of $1.5\sqrt{\pi }R_{j}/\tau$;^[^ ^39^ ^]^ the term $2\pi R_{j}^{3}$ in the equation denotes the initial volume of the bulge. This scaling analysis predicts the daughter drop radius *R_d_* ≈ 1.65*R_j_*, which is close to the linear fitting in Figure 2c.

Figure 2d reports the final size distribution of jet drops obtained from the impact of large drops (*R*_0_ ≈ 1.0 mm). *R_d_* varies from ≈ 40 to ≈ 500 µm, corresponding to a volume spanning over three orders of magnitude from ≈ 250 pL to ≈ 500 nL. The drops have approximately constant sizes at low and high Weber numbers (We≲6 and We>∼15), while another power‐law correlation, *R_d_* ∝*We* ^3/2^, is found at intermediate Weber numbers. Similar trends were also identified for larger drops with *R*_0_ ≈ 1.5 and 2.0 mm (Figure S6, Supporting Information), though at the same Weber number *R_d_* is slightly larger. We performed a scaling analysis on the jet dynamics to understand this dependence. During the emission of an inertial jet, the governing equation describing the free‐surface flow reads^[^ ^34^, ^40^ ^]^

(2) $$\rho \left(V_{t}+V\cdot \nabla V\right)+\gamma \nabla \left(\nabla_{s}\cdot n\right)=0$$

where *V* and **n** are the velocity and the normal vector at the jet surface, respectively; subscripts *t* and *s* denote the partial derivative with respect to time and the surface, respectively. The inertial and convective terms can be scaled as $|\rho V_{t}|∼|\rho V\cdot \nabla V|∼\rho V_{j}^{2}/L$, and the interfacial force as $|\gamma \nabla (\nabla_{s}\cdot n)|∼\gamma /R_{max}^{2}$, where the characteristic length *L* should be of the order of the capillary wavelength of the impinging drop $\gamma /\rho V_{0}^{2}$,^[^ ^34^ ^]^ while *R_max_* denotes the maximum spreading radius of the impinging drop on non‐wetting surfaces and it grows with the Weber number according to a power law $R_{max}∼R_{0}We^{1/4}$.^[^ ^30^, ^33^ ^]^ Substituting these terms into the non‐dimensional expression of Equation (2), we obtain $V_{j}\propto R_{0}^{-1/2}We^{-3/4}$, which was indeed observed in the experiments as illustrated in Figure S4c, Supporting Information. Moreover, the scaling analysis on the jetting dynamics suggests $V_{j}\propto R_{j}^{-1/2}$ for impinging drops at 6≲We≲15 (see Text S1 and Figure S4, Supporting Information), and the Rayleigh–Plateau instability induced jet drop formation obeys *R_d_* ≈ 1.65*R_j_* (see Figure 2c), and thereby

(3) $$R_{d}∼R_{j}\propto V_{j}^{-2}\propto R_{0}We^{3/2}$$

The above correlation is consistent with the experimental observations in Figure 2d, and its rationality can be further confirmed by plotting *R_d_*/*R*_0_ as a function of *We*, which collapses all experimental data of impinging macrodrops with different radii onto one master curve, as displayed in the inset of Figure S6, Supporting Information.

### 2.2 Design and Construction of the Macrodrop‐Impact‐Mediated Microdispenser

**Figure 3:**
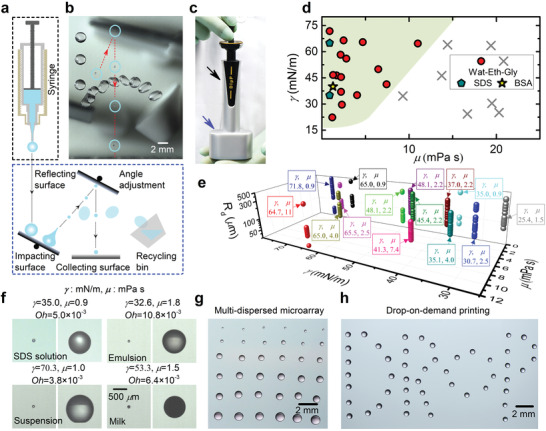
a) Sketch of the dispensing system containing a macrodrop generation unit and a microdrop impact monitoring chamber. b) Superposition of successive frames of an impinging water drop on the superamphiphobic surfaces. The produced jet drop is circled and its trajectory is drawn as the red dash line. c) Photograph of an assembled macrodrop‐impact‐mediated micropipette (DIµP) with a portable dimension. The black and blue arrows indicate the two functional units in (a). d) Phase diagram of drop generation of liquids with different viscosities (µ) and surface tensions (*γ*) using DIµP. The colored symbols in the green‐shaded region and grey crosses indicate the cases with and without jet drop generation, respectively. Wat‐Eth‐Gly, SDS and BSA indicate the water‐ethanol‐glycerol mixtures, the sodium dodecyl sulfate solutions and the bovine serum albumin, respectively. e) Jet drop radius *R*_d_ as a function of liquid surface tension *γ* and viscosity µ. f) Snapshots of produced jet drops of four types of complex liquids using the DIµP. The corresponding surface tension, viscosity, and Ohnesorge number $Oh=\mu /\sqrt{\rho R_{d}\gamma }$ are given. g) Deposited drop arrays on a hydrophobic surface with different volumes of ≈ 2.3 − ≈300 nL from the top row to the bottom row. h) Patterning a “DROP” word on a hydrophobic surface with ≈ 80 nL drops.

The impact‐stimulated jet drop formation provides a new concept to design an innovative, non‐contact drop dispenser by coupling it with the drop rebound behavior on non‐wetting surfaces.^[^ ^30^, ^34^, ^41^ ^]^ As sketched in **Figure** **3a**, the device consists of two integral parts: a top unit (framed in black) to generate microliter drops with controlled velocities, and a bottom chamber (framed in blue) to monitor the impact process and the produced jet drops. The impinging drop is released from a common injection needle (of a few hundreds of microns in diameter) attached to a syringe, which is secured on a position‐adjustable clamp to vary the release height. In the bottom chamber, the non‐wetting target surface is mounted on a tilting plate to enable controlled oblique drop impacts, whose small “daughter” drops would rebound and be redirected vertically downward via a second impact on another non‐wetting surface^[^ ^30^ ^]^ (reflecting surface) placed above, while the large “mother” drop is collected into a reservoir located aside. Moreover, superamphiphobic surfaces constructed by a fractal‐like network of hydrophobized silica shells^[^ ^42^ ^]^ are employed as the impinging targets. The fractal‐like network is a desirable structure to achieve superamphiphobicity,^[^ ^43^ ^]^ and thus repel diverse liquids with a wide range of surface tensions and viscosities. This impact scenario is theoretically achievable (Text S2 and Figure S7, Supporting Information), and has been practically realized by carefully adjusting the position of the target and reflecting surfaces with respect to the impinging drop, as demonstrated in Figure 3b and Movie S5, Supporting Information. The dispensing function can even be implemented and integrated into a portable device (Figure 3c and Figure S8, Supporting Information), which we call the macrodrop‐impact‐mediated micropipette (DIµP).

To assess the operational capabilities of the device, different water‐ethanol‐glycerol mixtures were tested. Our microdispenser can dispense low‐viscosity liquids with μ≲10mPas (Figure 3d). Higher viscosity would damp the capillary wave^[^ ^34^, ^44^ ^]^ and thus suppress the jet emission during drop impact. The working surface tension ranges from ≈ 25 mN m^−1^ to ≈ 72 mN m^−1^ (Figure 3d), covering the surface tensions of most laboratory solvents, and the corresponding Ohnesorge number of these impinging drops is in the range of 3.4 × 10^−3^ − 1.4 × 10^−1^. Since the drop mass loss due to evaporation is negligible (Text S3, Supporting Information), the dispensing volume should be the same as that of the jet drop, and most working liquids for our device can be dispensed from picoliters to nanoliters range as shown in Figure 3e and Figure S9, Supporting Information, spanning over three orders of magnitude in drop volume. To dispense a liquid drop with a predefined size/volume or tune the drop size/volume, one only needs to simply adjust the releasing height of the impinging macrodrop according to the correlation between *R_d_* and *We* shown in Equation (3), or more clearly, to the dependence of *R_d_* on the releasing height as shown in Figure S10, Supporting Information (where that of several representative liquids are illustrated), if a certain injection needle is employed. The dispenser is capable of dispensing not only simple and compound liquids, but also complex fluids including aqueous surfactant solutions, colloidal suspensions and emulsions (Figure 3f). Moreover, monodispersed and multidispersed drop arrays and patterns have been deposited on target surfaces by using a motor‐driven slide with a maximum drop generation frequency of ≈ 3 Hz (Figure 3g,h and Figure S11, Supporting Information), which is determined by the hydrodynamics of dripping (and Text S4, Supporting Information). This dispensing efficiency is much lower than that of other dispensing methods employing the hydrodynamic jetting (up to several tens of kHz^[^ ^21^ ^]^), but is comparable to that of the dip‐pen nanolithography (only several Hertz,^[^ ^13^, ^45^ ^]^ Table S2, Supporting Information).

Compared with most existing dispensing techniques,^[^ ^12^, ^13^, ^22^, ^23^, ^24^, ^26^, ^45^ ^]^ our macrodrop‐impact‐mediated dispenser does not require any complex external controlling systems to implement the dispensing function and the tuning of the drop size can be easily achieved by adjusting the releasing heights of the impinging macrodrops without changing the dispensing nozzle. The simple drop impact platform can be assembled using mechanical components fabricated by additive manufacturing (Figure S8, Supporting Information), and the fabrication cost of the portable micropipettes is reduced by at least a factor of ≈ 40 compared with commercial dispensers (Figure S12, Supporting Information). For industrial production, the cost could be further reduced. The good repeatability and robustness of the jetting^[^ ^30^, ^31^ ^]^ enables precise control of fluid dispensing volume with a standard deviation of less than 15% from the mean value (Figure S13, Supporting Information). However, because the dispensing function is realized via two impact processes (Figure 3a), the maximum offset distance of the microdrop location is estimated to be ≈ 200 − ≈500 µm depending on the sizes of the dispended microdrops, which is apparently larger than that of the ink‐jet printing (of the order of 10 µm)—a commercial technique that has been invented, developed, and optimized for several decades.^[^ ^21^ ^]^ Nevertheless, in the first phase of principle investigation and technique development, which is the main goal of this work, we have demonstrated that our dispensing technique can pattern microdrop arrays with an acceptable positioning precision, as shown in Figure 3g and Figure S11, Supporting Information. The positioning precision would be further improved by employing additional control platforms such as the external electric field,^[^ ^46^ ^]^ which is beyond of the scope of the current work.

### 2.3 Fluid Dispensing in Modern Diagnostics and Manufacturing

**Figure 4:**
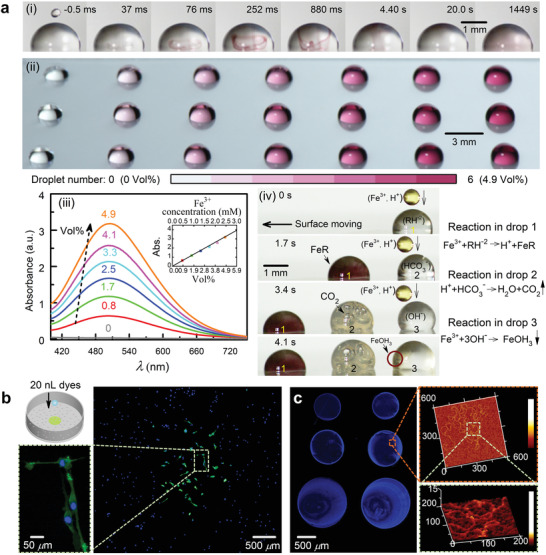
a) Controlled reaction of iron(III) and sulfosalicylic acid in sessile drops: i) coalescence and reaction of a 34 nL iron(III) drop with a 4 µL acid drop; ii) sessile drop arrays of pure sulfosalicylic acid and acid reacted with 1–6 iron(III) microdrops, where the iron(III) volume percent in the sessile drops ranges from ≈ 0.8% to ≈ 4.9%; iii) spectrophotometric results of the reacted drop arrays; iv) three different chemical reactions induced by successive reagent microdispensing. The inset plots the absorption peak at ≈ 506 nm as a function of Fe^3 +^ volume percent and Fe^3 +^ concentration. b) Schematic (top left) and results of cell staining. c) Left, DNA patterns after evaporating 4, 10, and 22 nL drops from top to bottom, respectively. Right, high‐solution atomic force microscopy images of DNA chains.

Volumetric dispensing is an essential procedure for microchemistry, where precise handling of small quantities of chemical compounds determines the accuracy of the outcome of chemical reactions.^[^ ^47^ ^]^ We perform an experiment of the reaction between iron(III) and salicylic acid in microliter drops to demonstrate that our macrodrop‐impact‐mediated microdispenser is able to manipulate chemicals exhibiting non‐wetting properties on superamphiphobic surfaces with a nanoliter resolution. As displayed in **Figure** **4a** (i) and Movie S6, Supporting Information, ≈ 34 nL aqueous iron(III) solution (6.2 × 10^–3^ m, pH ≈ 2) was injected into a 4 µL sulfosalicylic acid drop (10 × 10^–3^ m, pH ≈ 2) deposited on a hydrophobic glass slide using DIµP. After coalescence, the reacted acid turns violet within ≈ 200 ms, and the colored complex mixes well with the residual acid after ≈ 3 min. In order to elucidate the quantity‐sensing of the complex‐forming reaction, 1 − 6 aqueous iron(III) drops (with volume varying from ≈ 34 to ≈ 204 nL) were added to the acid drop, and the resultant products shade from light to deep violet with increasing iron(III) volume percent from ≈ 0.8% to ≈ 4.9% (Figure 4a (ii); Movie S7, Supporting Information). The amount of the formed complex can be inferred more precisely by a spectrophotometric analysis, in which the absorption intensity increases with the added volume of iron(III) (Figure 4a (iii)). The linear increase of the absorption maxima at ≈ 510 nm with the cumulative iron(III) volume (inset of Figure 4a (iii)) further demonstrates the repeatability of the dispensing volume, and the possibility of using our non‐contact dispensing technique for high‐throughput chemical screening. Moreover, it is worthy pointing out that this technique also allows us to independently manipulate different chemical reactions in a single stepwise dispensing procedure, as shown in Figure 4a (iv) and in Movie S8, Supporting Information.

Along with the good compatibility for normal solvents and suspensions, our microdispensing method is also able to transfer liquid samples in biological analysis. As an example, we conducted a double cell stain experiment, in which the nuclei of mice cells were first dyed blue by adding 200 µL DAPI solution (10 × 10^–6^ m) into the cell‐culture medium using commercial micropipettes, while the cell membranes were successively dyed green by adding 20 nL DiO solution (10 × 10^–6^ m) using our DIµP (Figure 4b). Though the cell staining is a simple biological experiment, it has demonstrated that the small‐volume dispensing function of DIµP ensures the accurate handling and positioning of biological samples in a small region down to ≈ 2.5 mm^2^ (i.e., the area occupied by green‐dyed cells in Figure 4b), which could be beneficial for selective cell analyses.^[^ ^3^, ^11^ ^]^ Most existing dispensing techniques are compromised by the delivery of biological samples containing DNA molecules or cells, largely because of the contamination of the small‐dimension dispensing nozzles by the biological fluids.^[^ ^11^, ^26^ ^]^ This is a negligible issue in our dispensing technique in which low‐cost and common injection needles are used. Figure 4c shows DNA microarrays produced by depositing and evaporating DNA‐laden drops on mica substrates using DIµP (left panel), and high‐resolution images of the DNA chains acquired by atomic force microscopy (right panel). It is well known that the genotyping and gene expression profiling always proceed with comparative analyses of up to thousands of DNA spots on transparent supports like glass slides,^[^ ^8^, ^48^ ^]^ and thus our dispensing device could be used to pattern DNA spot arrays with diverse dimensions.

**Figure 5:**
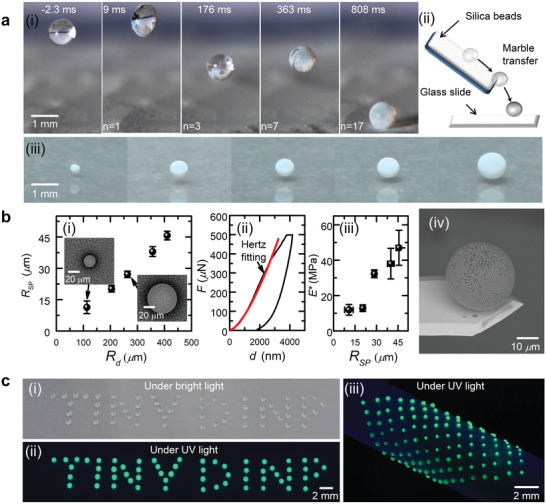
a) Fabrication of liquid marbles: i) Snapshots showing the impact of a water microdrop on hydrophobic powder of 5 µm‐diameter silica particles, forming a powder‐coated liquid marble; ii) sketch of the marble transfer; iii) liquid marbles with radii of ≈ 170 − ≈ 500 µm deposited on hydrophilic surfaces. b) Creation of supraparticles: i) radii of formed supraparticles *R_SP_* as a function of the dispensing volumes of the aqueous colloidal solution *R_d_*; ii) representative force–distance curve acquired in the nanoindentation measurements on a 20 µm‐radius supraparticle and the red line is the fit of the elastic regime using the Hertz model; iii) effective Young's modulus E* versus the radius of the supraparticle *R_SP_*; iv) SEM image of a colloidal probe made by attaching a 16 µm‐radius supraparticle. c) Light‐emitting displays made of ≈ 110 nL drop arrays on a glass substrate (i,ii) and a soft parafilm (iii) after being exposed to bright light and ultraviolet light of 365 nm wavelength.

In microfluidic applications, drop transport frequently occurs on solid surfaces and the strong liquid–solid adhesion inevitably leads to liquid deposition after removing the drop.^[^ ^49^ ^]^ One plausible strategy to overcome this issue is to encapsulate the liquid drop with hydrophobic particles, and thus turning it into a free flowing powdered material, that is, the so‐called “liquid marble.”^[^ ^50^ ^]^ The sequence of images in **Figure** **5a** (i) and Movie S9, Supporting Information, shows the formation of a liquid marble by injecting a water drop from the DIµP into a hydrophobic powder of 5 µm‐diameter particles, which would rebound many times and eventually gets itself encapsulated. Thanks to the wide volume tunability of our dispensing system, we have prepared liquid marbles with radii of ≈ 170 − ≈500 µm, which can roll off easily and be transferred onto hydrophilic glass slides in a non‐wetting state (Figure 5a (ii,iii)). Transparent liquid marbles of similar sizes were also fabricated using the same strategy with 100 nm‐diameter silica particles as displayed in Figure S14, Supporting Information.

To further highlight the potential application of the DIµP in material manufacturing, we fabricated porous supraparticles via evaporation‐induced self‐assembly. A drop of an aqueous suspension of silica microspheres (1 µm in diameter) was delivered from the microdispensing system onto a superhydrophobic surface. After water dried out, a spherical microstructure constructed of closely packed microspheres was left behind. By tuning the size of the colloidal drops (from ≈ 100 to ≈ 400 µm), supraparticles with radii of ≈ 11 − 45 µm were obtained (Figure 5b (i)). Notably, highly ordered crystalline patches are present on the surface of these microstructures, particularly in the region close to their bottom and on small supraparticles. The formation of these crystalline patches is attributed to the local competition between the water vapor diffusion into the air and the particle diffusion in the drop.^[^ ^51^ ^]^ Nanoindentation measurements were performed to characterize the mechanical properties of these porous micromaterials (Figure 5b (ii); Figure S15 and Text S5, Supporting Information), which indicate that their effective Young's moduli are on the order of several tens of MPa and increase with the size, as illustrated in Figure 5b (iii). Because of the small contact area and thus the low adhesion between the formed supraparticles and the supporting superhydrophobic surface, these supraparticles can be easily manipulated in further material processing. Figure 5b (iv) shows a colloidal probe which was obtained by attaching such a supraparticle to a cantilever. Our fluid dispenser has also been employed for patterning other functional materials or salt crystallization through drop evaporation (Figure S16, Supporting Information).

High‐resolution dispensing techniques, such as ink‐jet printing, have clear advantages of low‐cost, high precision, easy processing, and low material consumption, and thus are also used for small‐scale device manufacturing.^[^ ^2^, ^21^ ^]^ As a final demonstration, we fabricated a UV‐activated display on a glass substrate using small drop arrays that were generated by DIµP. The display was printed with pre‐defined drop patterns, and exhibited a shadowy image under white light (Figure 5c (i)), but a very good image quality under UV light (Figure 5c (ii)). As the dispensing technique is compatible with any substrates, drop pattern printing can even be carried out on paper‐like soft substrates (Figure 5c (iii)), a promising pathway to make wearable photo or electronic devices.

## 3 Conclusion

By exploiting the jetting phenomenon occurring in the impact of macroscopic microliter drops on nonwetting surfaces, we propose and design a facile non‐contact approach to dispense single small drops on demand. The monodispersed drops are triggered and successively generated through the breakup of a singular jet, which is formed by the collapse of the air cavity resulting from drop impact. Despite this strategy being only adaptable to dispense low‐viscosity liquids, the operational surface tensions cover a wide range of organic and inorganic solvents in the laboratory, and the produced drop volume can be tuned from picoliters to nanoliters. In comparison with most contact and non‐contact dispensing methods,^[^ ^12^, ^13^, ^17^, ^20^, ^22^, ^23^, ^25^, ^26^, ^27^ ^]^ our technique requires neither small‐scale dispensing tips/nozzles involving complicated microfabrication procedures nor large, expensive operating systems, and the rather simple impact platform enables us to fabricate a compact, portable pipette with a nanoliter resolution. The microdrop generation frequency of our dispenser is limited by the hydrodynamics of macrodrop dripping, and is apparently lower than that of other dispensing techniques developed based on the jet formation and instability, but is comparable with that of most contact fluid dispensing approaches. The simple common injection needles used allow transferring not only normal suspension and emulsion materials, but also biological fluids containing DNA molecules or cells, which would otherwise cause contamination in commercial non‐contact dispensers such as ink‐jets.^[^ ^21^ ^]^ These unique features of our dispenser are promising for some real‐world applications requiring precise liquid handling with a low dispensing frequency.

## 4 Experimental Section

### Materials

Four smooth (surface I–IV) and five structured surfaces (surface V–IX) with different wetting properties were prepared in the experiment. Silicon wafers were cleaned with isopropanol and then ethanol in an ultrasonic bath for 5 min each. After rinsing in water and drying with purified nitrogen, they served as hydrophilic surfaces (surface I). Treating silicon wafers in oxygen plasma (PDC‐002, Harrick plasma, USA) for 5 min and then silanizing with hexamethyldisilazane in the vapor phase at 75 ^○^C for 10 h, weakly hydrophobic surfaces (surface II) were obtained. Intermediate hydrophobic surfaces (surface III) were prepared by silanizing plasma‐treated silicon wafers with 1H, 1H, 2H, 2H‐perfluorodecyl‐thiethoxysilane. Another type of flat hydrophobic surfaces (surface IV) were 1 mm thick polydimethylsiloxane (PDMS, Sylgard 184, Dow Corning, Wiesbaden, Germany) substrates with monomer/cross‐linker ratio of 10: 1, which were coated on glass slides and cured at 75 ^○^C for 12 h. Micro‐pillared PDMS substrates (surfaces V and VI) were fabricated using soft lithography, and the corresponding widths of the square pillars are 5 and 20 µm, and interpillar distances are 20 and 100 µm, respectively, while their heights were kept at 2 µm. To further enhance surface hydrophobicity, a thin layer of hydrophobic nanoparticles (Ultra Glaco, Soft 99 Co., Japan) and Ultra‐Ever Dry (Ultra Tech International, Inc., USA) were sprayed onto the micro‐pillared PDMS surfaces (surface V), resulting in two superhydrophobic surfaces (surfaces VII and VIII). The superamphiphobic surface (surface IX) was prepared by depositing a fractal‐like network of hydrophobized silica shells on glass substrates using candle soot as a template.^[^ ^42^ ^]^ Scanning electron microscopy images of these structured surfaces (surface V‐IX) are shown in Figure S2, Supporting Information, and contact angles of 4 µL water drops on all surfaces are summarized in Table S3, Supporting Information.

Hydrophobized silica particles with diameters of 5 and 100 nm were purchased from Suzhou Research Materials Microtech and used for liquid marble preparation. Ethanol (>99.7%), glycerol (>99.0%) and ethylene glycol (>99.0%) were purchased from Sinopharm Chemical Reagent, and mixed with pure water (18.4 MΩ cm, Millipore Synergy, Darmstadt, Germany) to prepare liquids with viscosities of 0.9 − 20.3 mPa s and surface tensions of 22.3 − 71.8 mN m^−1^. Dodecane, cetane, methylene chloride, and sodium dodecyl sulfate (SDS) were obtained from Aladdin and used as received. BSA was purchased from Zhejiang Tianhang Biotechnology CO., LTD. Aqueous colloidal suspension containing 5 µm‐diameter and 1 µm‐diameter polystyrene particles (Suzhou Research Materials Microtech) was diluted from ≈ 0.02 to ≈ 8 × 10^−4^ g mL^−1^ and from ≈ 10^−2^ to ≈ 10^−4^ g mL^−1^, respectively. Aqueous emulsion was prepared using hexadecane and its mass concentration was 10 wt%, while 0.025 wt% SDS was added as the stabilizer for the water/oil system. The physical properties of these liquids and their contact angles on the superamphiphobic surface (surface IX) are reported in the Table S4, Supporting Information.

### Drop Impact Tests

Liquid drops were released from blunt needles with diameters of 0.24, 0.70, and 2.0 mm using a syringe pump. They were accelerated under the gravity and impacted on the solid surface placed underneath. The impact process was recorded using a high speed camera at 60000 − 340000 fps. For each experimental parameter, at least three impact tests were performed and analyzed. To characterize the volume controllability of the DIµP, we measured the volumes of 50 microdrops of water, 60 wt% glycerol‐water mixture, 0.14 wt% SDS solution and milk at two Weber numbers, and analyzed the correponding volume distribution, respectively.

### Light Emitting Display

The fluorescent solution was prepared by dissolving 0.02 mg mL^−1^ 8‐hydroxypyrene‐1,3,6‐trisulfonic acid trisodium salt into 5 wt% glycerol‐water mixture. 110 nL drop arrays of such solution were deposited on a glass slide using our macrodrop‐impact‐mediated fluid microdispensing technique with the assistance of a motor‐driven slide. The display emits green light under the illumination of 365 nm ultraviolet light.

### Chromogenic Reaction

6.2 mm aqueous solution of iron(III) chloride (Sinopharm Group Co., China) was first prepared, and then hydrochloric acid was added to inhibit the hydrolytic reaction until a pH value of 2 was reached. The same procedure was also applied to obtain 10.0 × 10^–3^ m aqueous solution of sulfosalicylic acid (Sinopharm Group Co., China.) with pH = 2. 4 µL acid drop arrays were patterned on a hydrophobic glass substrate using a commercial pipette (Mettler‐Toledo RAININ, Columbus, Ohio, USA), and ≈ 34 to ≈ 204 nL Iron(III) chloride solution was successively added to induce the following chemical reaction

(4) $$Fe^{3+}+RH^{-2}\to H^{+}+\mathrm{FeR}$$

where R represents the sulfosalicylic acid ion. The formed FeR complex can be inferred from the absorbance spectra using a spectrophotometer (Shimadzu UV‐2550, Japan), where an intensity peak was observed at the wavelength of *λ* ≈ 506 nm.

### Manipulating Different Chemical Reactions by Successive Microdispensing

0 mm aqueous solution of Iron(III) chloride with pH = 2 was prepared and served as the dispensing reagent. Three sessile drops (with a volume of 4 µL) of 10.0 × 10^–3^ m aqueous solution of sulfosalicylic acid, 0.1 × 10^–3^ m aqueous solution of sodium bicarbonate, and 0.1 m aqueous solution of sodium hydroxide were deposited on a hydrophobic glass substrate with an interval of 4 mm. Then, 500 nL Iron(III) chloride solution was successively added to these three drops using our dispensing system, which induces the following chemical reactions: 1) Fe^3 +^ + RH^−2^ → H^+^ + FeR; 2) H^+^ + HCO_3_^−^ → CO_2_^↑^ + H_2_O; 3) Fe^3 +^ + 3OH^−^ → FeOH_3 ↓_.

### Double Fluorescent Staining

Mouse nerve cells were employed for the staining experiment. 500 µL cell suspension (with a concentration of ≈ 20 cells per microliter) was pipetted onto a 14 mm‐diameter glass plate, which was incubated at 37 ^○^C under 5% CO_2_ environment for 24 h. Then, 200 µL aqueous solution of 4′,6‐diamidino‐2‐phenylindole (10 × 10^–6^ m, DAPI, Roche, Germany) was added to mark cell nuclei, and the glass plate was incubated for another 7 min. After rinsing the cell‐attached plate in the phosphate buffer saline (PBS, Gibco, Thermo Fisher Scientific, Waltham, MA, USA), 20 nL aqueous solution of 3,30‐dioctadecyloxacarbocyanine perchlorate (10 × 10^–6^ m, DiO, Beyotime, China) was then introduced to mark cell membranes using our DIµP, and same post‐dyeing procedures were followed. The double stained cells were further sealed in antifade mounting medium (Beyotime, China) and characterized using an inverted fluorescent microscopy (ECLIPSE Ti‐U, Nikon, Japan).

### DNA Patterning and AFM Imaging

1 mg mL^−1^ double‐stranded lambda DNA (48.5 k base pairs, Thermo Fisher Scientific, Waltham, MA, USA) solution was first diluted to 2 µg mL^−1^ using Milli‐Q water (Synergy UV, Merck), and then stained by adding aqueous solution of Hoechst 33342 (the final concentration is ≈ 3 µg mL^−1^). To enhance the absorption of negatively charged DNA molecules, the freshly cleaved mica was immersed in the 10 × 10^–3^ m NiSO_4_ (Sinopharm Group Co., China) solution for 10 min. After rinsing in water and drying with purified nitrogen, 0.4, 10, and 74 nL DNA‐laden drops were deposited on the mica substrate using the DIµP. The DNA patterns after solvent evaporation were imaged under the inverted fluorescent Nikon microscope. High‐resolution images of DNA molecules were acquired using an atomic force microscope (AFM, Bioscope Resolve, Bruker) with the PeakForce tapping mode in air. The silicon nitride cantilever probes (SNL‐A, Bruker) with a spring constant of 0.61 N m^−1^, tip radius of 2.0 nm, and tip half angle of $20\pm 2.5^{\circ }$ were used. Prior to imaging, the spring constant and the deflection sensitivity of the cantilever were calibrated using the thermal noise spectrum. The minimum peak force of 100 pN for stable imaging was used and all images were captured at a resolution of 512 × 512.

### Nanoindentation Measurements and Analyses

Nanoindentation tests were conducted using the Hysitron TI 900 Triboindenter (Hysitron Inc., Minneapolis, USA), which is equipped with a low load QSM transducer and a diamond flat‐ended indenter tip with a diameter of 10 µm. After contacting the sample, the indenter tip was driven into the sample with a constant loading rate of 70 µN s^−1^ until a maximum load force of 500 µN was reached. The peak load was held for 2 s to make the indenter tip steady, and then an unloading process was followed with the same force rate in the loading process. The acquired force–distance curve in the early loading process was analyzed by the Hertz model to obtain the effective modulus.

### Statistical Analysis

The macrodrop impact experiments were performed at least three times for each experimental parameter and similar results were obtained. To assess the repeatability of the dispensing ability, 50 microdrops were generated and their volume distributions were fitted with the Gaussian function in OriginPro. All data were presented as the average values ± standard deviations of independent measurements.

## Conflict of Interest

The authors declare no conflict of interest.

## Author Contributions

L.C., X.D., and S.L. conceived the research and designed the experiments. L.C. supervised the research. S.L. performed the experiments and analyzed the data. D.W., L.Z., and Y.J. assisted in part of experiments. S.L. and L.C. built the analytic models. S.L., L.Z., Z.L., E.B., Z.Y., X.D., and L.C. interpreted the data. L.C. and S.L. wrote the manuscript with input from all authors.

## Supporting information

Supporting Information

Supplemental Movie 1

Supplemental Movie 2

Supplemental Movie 3

Supplemental Movie 4

Supplemental Movie 5

Supplemental Movie 6

Supplemental Movie 7

Supplemental Movie 8

Supplemental Movie 9

## Data Availability

The data that support the findings of this study are available from the corresponding author upon reasonable request.
