# Supplementary material for: Macrodrop‐Impact‐Mediated Fluid Microdispensing
Source: Adv Sci (Weinh). 2021 Jun 26;8(16):2101331. doi: 10.1002/advs.202101331 (PMC8373096; doi:10.1002/advs.202101331)
Supplement: Supplementary file 1 — Supporting Information [file ADVS-8-2101331-s004.pdf]

© 2021 Wiley-VCH GmbH

ADVANCED SCIENCE
Open Access

Supporting Information

for *Adv. Sci.,* DOI: 10.1002/advs.202101331

Macrodrop-impact-mediated Fluid Microdispensing

*Shiji Lin, Dehui Wang, Lijuan Zhang, Yakang Jin, Zhigang Li, Elmar Bonaccurso, Zili You, Xu Deng, and Longquan Chen**

Supporting Information

Macrodrop-impact-mediated Fluid Microdispensing

*Shiji Lin, Dehui Wang, Lijuan Zhang, Yakang Jin, Zhigang Li, Elmar Bonaccurso, Zili You, Xu Deng, and Longquan Chen**

S. Lin, Prof. L. Chen
School of Physics, University of Electronic Science and Technology of China,
Chengdu, Sichuan 611731, P. R. China
Email: lqchen@uestc.edu.cn

Dr D. Wang, Prof. X. Deng
Institute of Fundamental and Frontier Sciences, University of Electronic Science and Technology of China,
Chengdu, Sichuan 610054, P. R. China

L. Zhang, Prof. Z. You
School of Life Science and Technology, Center for Informational Biology, University of Electronic Science and Technology of China,
Chengdu, Sichuan 610054, P. R. China

Y. Jin, Prof. Z. Li
Department of Mechanical and Aerospace Engineering, The Hong Kong University of Science and Technology,
Clear Water Bay, Kowloon, Hong Kong SAR, P. R. China

Dr. E. Bonaccurso
AIRBUS Central R & T, Materials X,
Munich 81663, Germany

Text S1: Jet dynamics in drop impact

As inferred by Figure 1b, a thin jet is generated through the axial implosion of the air cavity (Figure 1b), which proceeds according to [1, 2] $R_c = At^{0.5}$, where A is a coefficient and t is time. At the onset of jetting, the liquid flowing across the cavity surface, $2\pi R_0 R_c \dot{R}_c$, is transferred to that of the growing jet, $2\pi R_c^2 V_j$, which yields $V_j \propto R_c^{-2}$. In addition, the kinetic energy of the implosion flow, which scales with $\pi\rho R_0 \dot{R}_c^3 R_c$, is converted into the kinetic energy of the jet, which is estimated as $\pi\rho \mathrm{V}_j^3 R_j^2$. These scalings result in $V_j \propto R_j^{-1}$, which is in good agreement with the experimental data. On the other hand, the thick jet formation (**Figure 1c**) bears some similarity with the jet emission in bubble bursting,[3, 4] which originates from the focusing of the capillary wave. The rather small Ohnesorge number ($Oh = \mu/(\rho R_j \gamma)^{0.5} \sim O(10^{-2})$) of the jetting flow implies that the dynamics is governed by inertial and capillary forces, and the dimensional analysis predicts $V_j \propto \sqrt{\gamma/\rho R_j} \propto R_j^{-0.5}$, which also matches the experimental observations.

Text S2: The design principle of DIμP

Figure S7a plots the radii of the produced jet drops as a function of the normal Weber numbers ($We_n = \rho R_0 V_0^2 \cos\alpha^2/\gamma$) with respect to the inclined superhydrophobic surfaces of three different tilting angles (α). Clearly, all data collapse onto one master curve with three distinct regions as defined in the manuscript, suggesting the repeatability of the phenomenon of jet drop formation during impact. Thus, we can design a microdispenser via the sketched oblique impact in the manuscript with an impact velocity of $0.4 - 1.2$ m/s (macrodrop releasing height of $8.0 - 73$ mm), and the produced drop volume ranges from picoliters to nanoliters. To direct the produced jet drop into the vertical direction, another reflection superhydrophobic surface is employed. By comparing the incident angles (α_1 and α_2) and the reflection angles (α_1' and α_2') illustrated in Figure S7b, we found that they are very close to the inclined angle of the surface, implying that the oblique impact almost follows the law of

reflection. Moreover, to ensure that the jet drop can be collected out of the device, it should not coalesce with the mother drop rebounding from the target surface. Therefore, the time taken for the jet drop and the mother drop to travel to the joint point (i.e. point C in Figure S7c) of their trajectories (indicated by the blue and red lines, respectively) should not equal. Since the velocity of the jet drop is the same as the jet velocity V_j, its flying time to point C can be estimated as

$$t_1 = \frac{(S_1 + S_2)}{V_j} \tag{1}$$

where $S_1 = d/\cos\alpha$, $S_2 = S_1 \sin\beta - l_y$, $\beta = \frac{\pi}{2} - 2\alpha$, and d is the distance between the target and reflection surfaces in parallel. On the other hand, the time for the flying mother drop can be calculated as

$$t_2 = \frac{l_x}{V_{0x}} \tag{2}$$

where l_x is the horizontal distance between point C and point A, and $V_{0x} = V_0 \cos\beta$ is the horizontal velocity component after impact. Another correlation is from the horizontal projectile motion, which describes the deceleration of the mother macrodrop by gravity, $l_y = V_{0y} t_2 - \frac{1}{2} g {t_2}^2$ with l_y being the rising height of the macrodrop and g being the gravitational acceleration. The coalescence can be avoid if $t_1 \neq t_2$ and thereby

$$d \neq \frac{2\left(V_0 V_j - {V_0}^2\right)\cos\alpha}{\mathrm{g}} \tag{3}$$

Taking typical parameters of the impact events ($V_0 = 0.7$ m/s, $V_j = 4$ m/s and $\alpha = 15^\circ$), a separation distance of ~13 mm is obtained.

Text S3: Theoretical analysis for the effects of evaporation on the produced jet drop mass

We consider the evaporation of a flying drop with a velocity V_0 under the laboratory conditions with temperature $T = 298$ K and a relative humidity of $RH = 60$ %. The rate of mass loss due to evaporation can be described as [5, 6]

$$-\frac{\mathrm{d}m}{\mathrm{d}t} = -4\pi R^2 DG \frac{dC}{dR} \tag{4}$$

where $m = 4\pi\rho_d R_t^3/3$ is the drop mass with ρ_d and R_t being the density and the radius of the drop at time t, R is the radial distance from the center of the drop, D is the diffusion coefficient, C is the concentration of the vapor, $G = 1 + 0.141Re^{0.6}$ is a coefficient characterizing the influence of drop motion on molecular diffusion, $Re = \rho R_d V_d/\mu$ is the Reynolds number with μ being the liquid viscosity. Applying the boundary conditions that C equals to the concentration of saturated vapor at $R = R_t$ and $C = C_\infty$ at $R = \infty$, Eq. [4] can be simplified to

$$-R_t \frac{\mathrm{d}R_t}{\mathrm{d}t} = \frac{DG(c_s - c_\infty)}{\rho_d} \tag{5}$$

Integrating the above equation yields

$${R_d}^2 - {R_t}^2 = \frac{2DG(c_s - c_\infty)}{\rho_d} t \tag{6}$$

where R_d is the initial drop radius. The mass loss ratio η can be determined as

$$\eta = 1 - \frac{\left({R_d}^2 - \frac{2DG(c_s - c_\infty)}{\rho_d} t\right)^{\frac{3}{2}}}{{R_d}^3} \tag{7}$$

Taking typical physical values of a jet drop with $R_d = 50\ \mu\mathrm{m}$ ($c_s = 0.023\ \mathrm{Kg/m^3}$, $c_\infty = 0.014\ \mathrm{Kg/m^3}$, $D = 2.6 \times 10^{-5}\ \mathrm{m^2/s}$, $V_d \approx 8.0\ \mathrm{m/s}$, $G \approx 6.6$, $\rho_d = 1000\ \mathrm{kg/m^3}$ and $t \approx 0.006$ s), we estimated a maximum mass change ratio of $\eta \approx 1.1 \times 10^{-2}$ for water, which suggests that the evaporation of produced jet drops can be neglected.

Text S4: Estimation of the maximum generation frequency of jet drops

We produce jet drops by impinging microliter macrodrops on non-wetting surfaces. Therefore, the maximum rate of drop generation should be determined by the dripping frequency. It has been known that the critical Weber number of the injection flow We_c, above which the transition from dripping to jetting occurs, takes the form [7]

$$We_c = 4\frac{Bo_{out}}{Bo_{in}}[1 + KBo_{out}Bo_{in} - ((1 + KBo_{out}Bo_{in})^2 - 1)^{1/2}]^2 \quad (8)$$

where Bo_{out} and Bo_{in} are the Bond numbers ($Bo = (\frac{\rho_d g d^2}{2\gamma})^{1/2}$) defined with the inside (d_{in}) and outside (d_{out}) radius of the injection needle with g and γ being the gravitational acceleration and liquid surface tension, respectively; K is a coefficient of ~ 0.37. The calculated We_c is ~ 15.2 ($d_{in} \approx 60\ \mu\text{m}$ $d_{out} = 240\ \mu\text{m}$) and thus the maximum injection speed is $V_m \approx 4.3\ \text{m/s}$. The formed pendant drop at the needle tip will fall when its volume reaches a critical value Ω_c ($\sim 4.2\ \mu$L for water) so that the gravity exceeds the capillary force [8]. The shortest time to generate an impinging drop can be estimated as $\tau_{drip} = 4\Omega_c/\pi d_{in}^2 V_m$, which is $\sim 345\ \text{ms}$. Thus, the maximum frequency of drop generation of our dispenser is about 3 Hz.

Text S5: Mechanical characterization of supraparticles

The mechanical properties of the assembled supraparticles were characterized using the TI900 TriboIndenter and a flat diamond probe with a tip diameter of 10 μm was employed in the measurements. As illustrated in Figure S10a, in each test the tip was pushed on the sample until a maximum loading force of $500\ \mu$N was reached, and then it was retracted back, during which the tip load and indentation distance were recorded. A representative force-distance curve is shown in Figure S10b. Since the indentation distance ($d \sim 4\ \mu$m) is shorter than the tip diameter, we model the supraparticle-tip contact as an elastic contact between a sphere and a rigid flat substrate, and thus the relationship between the loading force F and indentation distance d can be described by [9, 10]:

$$F = E^* \times \frac{4}{3} R^{1/2} d^{3/2} \tag{9}$$

where R is the radius of the supraparticle and E^* is the effective Young's modulus. To obtain the effective Young's modulus, we fitted the elastic deformation regime ($d \leq 1\ \mu$m) in the force-distance curve, as shown in Figure S10b.

Supplementary Figures

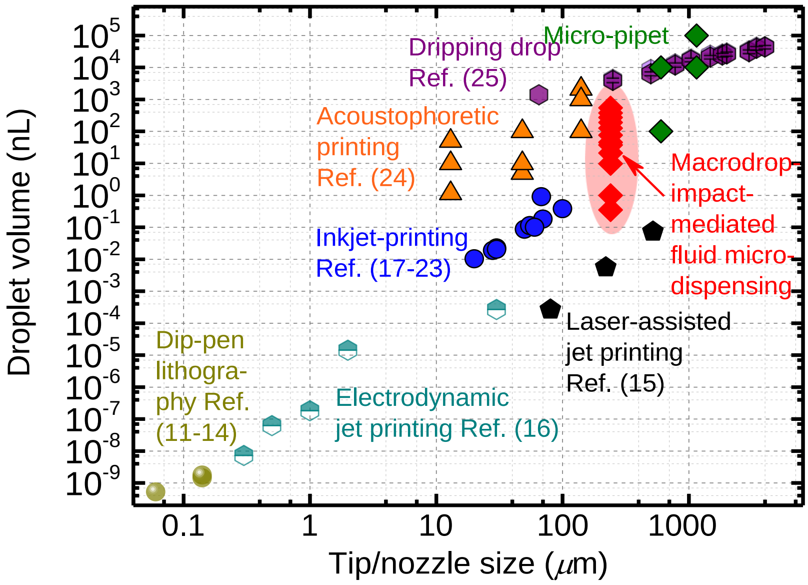

Figure S1. Comparison of drop volume range of the proposed macrodrop-impact-mediated microdispensing strategy with diverse dispensing approaches reported in the literature, including dip-pen nanolithography using sharp atomic force microscope tips,[11-14] laser-induced hydrodynamic jetting,[15] electric-field-induced hydrodynamic jetting,[16] inkjet printing,[17-23] acoustophoretic printing,[24] and liquid dripping employing commercial micropipetes or common injection needles.[25] The data are adapted from relevant references or from datasheet of the commercial products.

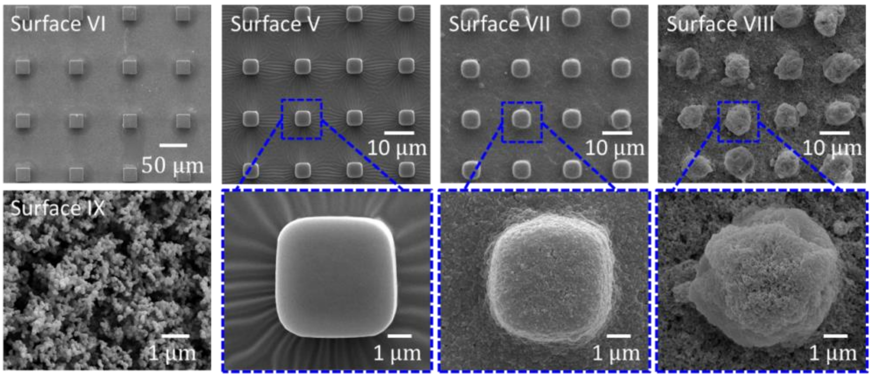

Figure S2. Scanning electron microscopy (SEM) images of structured surfaces (i.e. surfaces V-IX in **Table S2**).

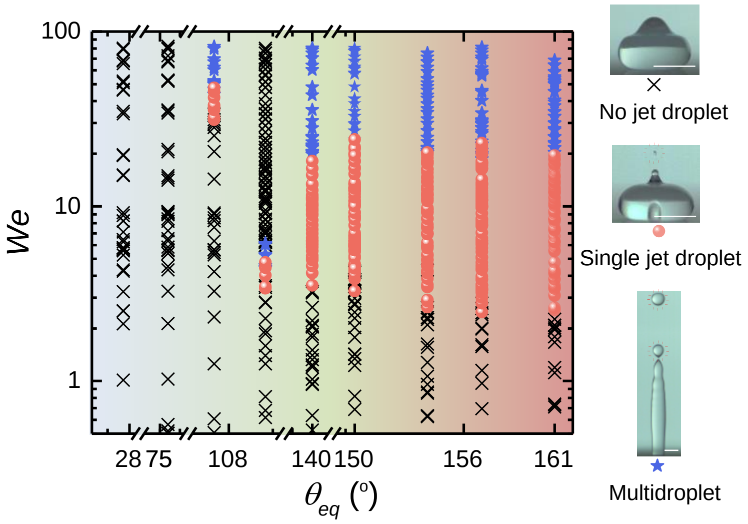

Figure S3. Jet drop formation versus the Weber number and surface wettability. The generation of a single jet drop only occurs on solid surfaces with $\theta_{eq} \gtrsim 106°$ and in a narrow range of We, which shows a nontrivial dependence on the surface wettability. While on surface with $\theta_{eq} \approx 106°$, jet drop generation is observed at high Weber numbers ($47 \gtrsim We \gtrsim 32$). On surfaces with $\theta_{eq} \gtrsim 113°$, jet drop generation takes place in the low Weber number regime and the lower We threshold is always around ~3.0. In contrast, the upper We threshold, above which multi-drops are produced or jet instability is suppressed, increases from ~5.0 on the surface with $\theta_{eq} \approx 113°$ to ~20.0 on surfaces with $\theta_{eq} \gtrsim 140°$. In general, single jet drop generation is quite robust on very hydrophobic surfaces with $\theta_{eq} \gtrsim 140°$.

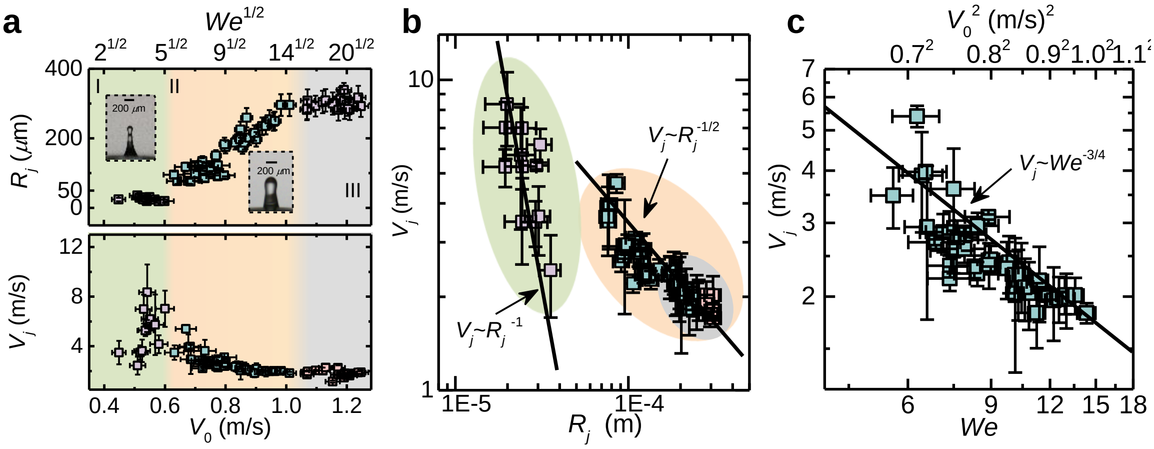

Figure S4. (**a**) Variation of the jet radius R_j (top) and the jet velocity V_j (bottom) as a function of the impact velocity V_0. (**b**) Variation of the jet velocity V_j versus normalized jet size R_j/R_0 , where R_0 is the initial drop radius. The green, orange and gray-shaded regions in (**a**) and (**b**) indicate different regimes for satellite drop generation. (**c**) Log-log plot of the jet velocity V_j as a function of the Weber number We at impact region with $6.0 \lesssim \mathrm{We} \lesssim 15$ for impinging macrodrops with radii $R_0 \approx 1.0$ mm.

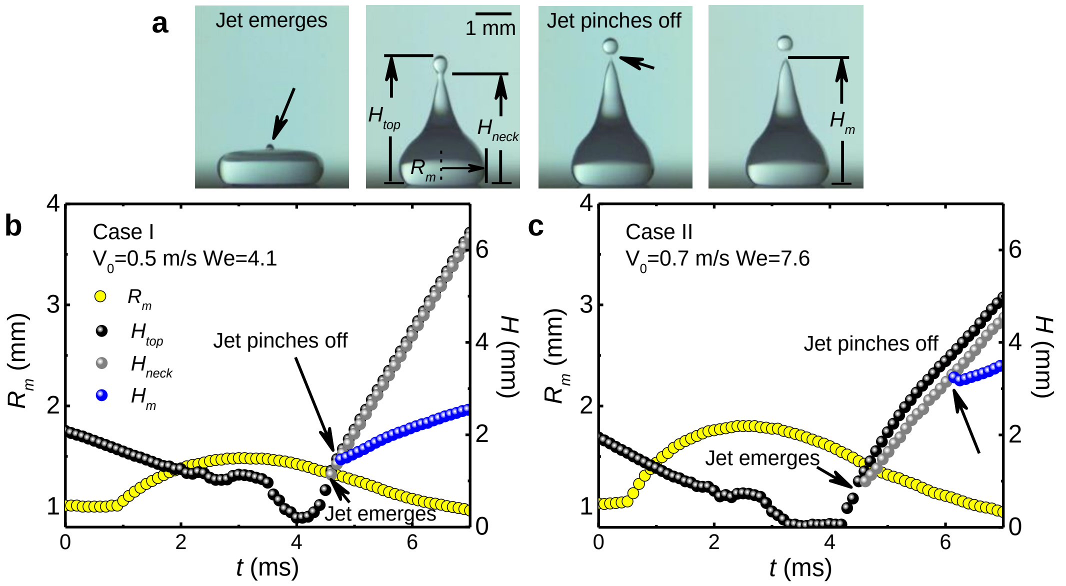

Figure S5. (**a**) Selected snapshots of impinging drop at $We \approx 7.6$**.** (**b-c**) the variation of the maximum curvature radius (R_m) of the impinging drop, the drop height (H_{top}) of the impinging drop, the height at which the necking occurs H_{neck}, and the height of the impinging drop after the jet drop detaches H_m. The Weber number for the impact events in (**b**) and (**c**) are 4.1 and 7.6, respectively. The slope of H_{neck} is almost the same as that of H_{top} after jet drop generation, suggesting that the jet drop is shot out with the jet velocity while the mother jet contracts back as the slope of H_m is much smaller.

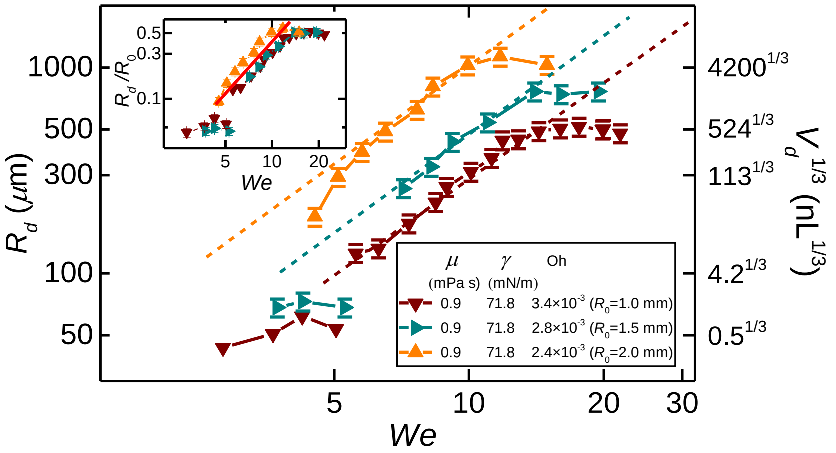

Figure S6. Jet drop radius R_d and volume V_d versus the Weber number We in log-log coordinates for impinging water drops with $R_0 \approx 1.0$, 1.5 and 2.0 mm. The power law correlation $R_d \propto We^{3/2}$ is identified for all macrodrops at intermediate Weber numbers $6 \lesssim \text{We} \lesssim 15$. The inset shows the plot of R_d/R_0 versus We.

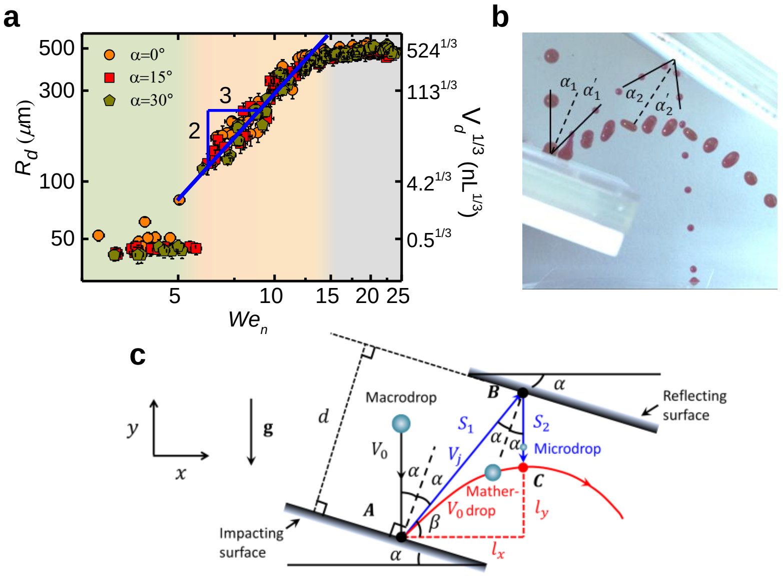

Figure S7. Jet drop generation in oblique macrodrop impact. (**a**) Produced jet drop radius R_d as a function of the normal Weber number We_n on three inclined surfaces with $\alpha = 0° - 30°$. (**b**) Superposition of successive frames of an impinging water drop on the superamphiphobic surfaces with $\alpha = 15°$ and $We_n \approx 13$. α_1 and α_1' are the incident and the reflection angles for the impinging macrodrop, while α_2 and α_2' denote the incident and reflection angles of the impinging jet drop on the reflection surface, respectively. (**c**) The sketched travel trajectories of the jet drop and the mother macrodrop.

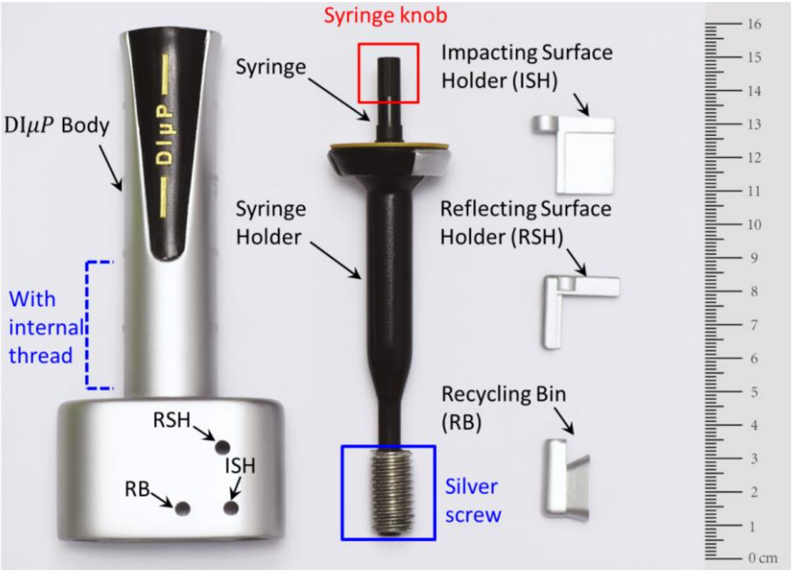

Figure S8. Mechanical components of DIμP. Mechanical components used to assemble the DIμP in **Figure 3c**. Macroscopic microliter drops can be generated by turning the syringe knob (denoted by the red box), and the release height can be adjusted by driving the steel screw (denoted by the blue box).

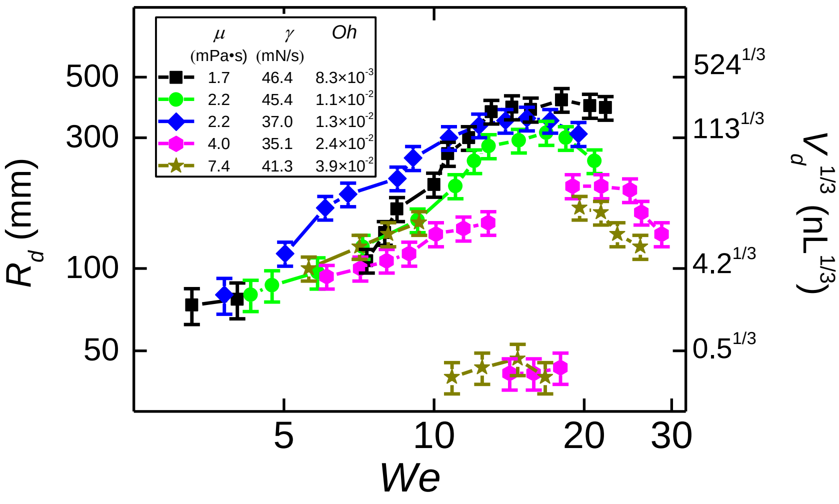

Figure S9. Jet drop radius R_d and volume V_d versus the Weber number We in log-log coordinates for impinging macrodrops with an Ohnesorge number Oh ranging from 8.3×10^{-3} to 3.9×10^{-2}.

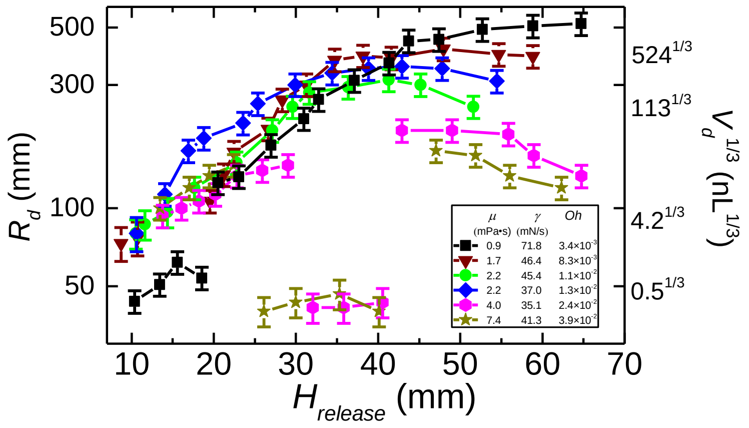

Figure S10. Jet drop radius R_d and volume V_d versus the releasing height $H_{release}$ of the impinging macrodrops, which were generated via an injection needle with an outer diameter of 240 μm, for diverse liquids an Ohnesorge number Oh ranging from 3.4×10^{-3} to 3.9×10^{-2}.

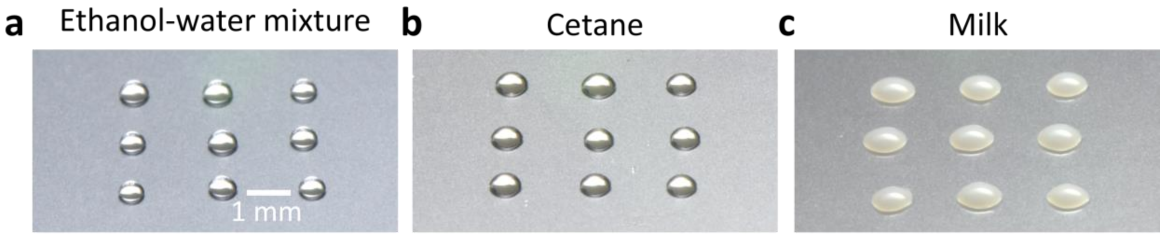

Figure S11. Printed microdrop arrays of 20 wt% ethanol-water mixture with $\mu = 1.4\ \mathrm{mPa \cdot s}$ and $\gamma = 38.6\ \mathrm{mN/m}$ (**a**), cetane with $\mu = 3.1\ \mathrm{mPa \cdot s}$ and $\gamma = 27.1\ \mathrm{mN/m}$ (**b**) and milk with $\mu = 1.5\ \mathrm{mPa \cdot s}$ and $\gamma = 53.5\ \mathrm{mN/m}$ (**c**) on hydrophobic glass slides.The corresponding drop volumes are 270 nL, 380 nL and 510 nL, respectviely.

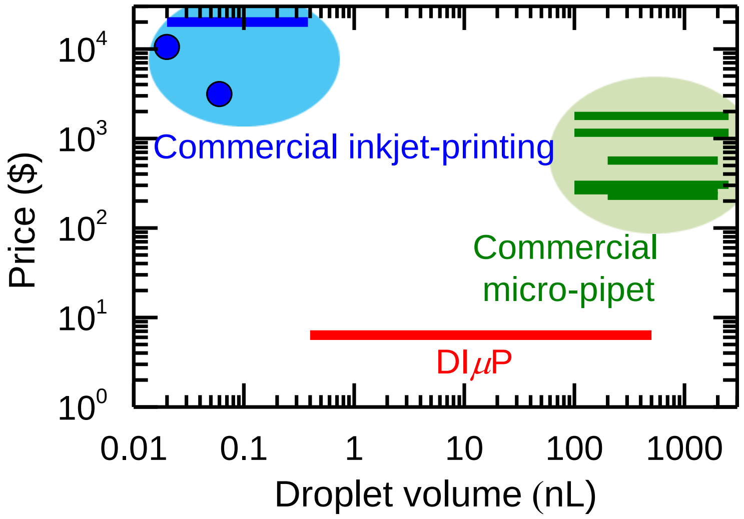

Figure S12. Comparison of the prices of diverse commercial dispensers as a function of the drop volume.

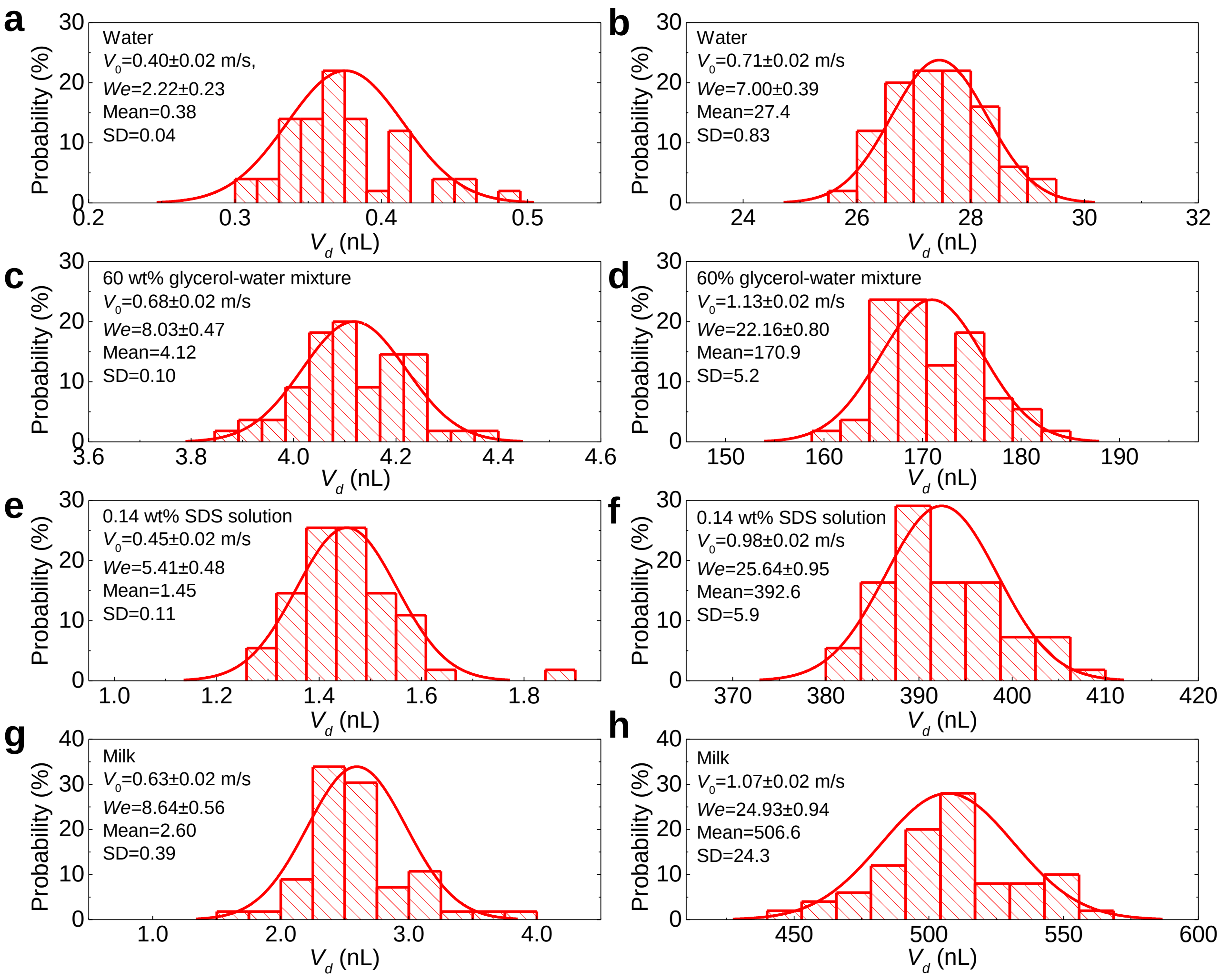

Figure S13. Volume distribution of produced microdrops of water with $\mu = 0.9\ \mathrm{mPa \cdot s}$ and $\gamma = 71.8\ \mathrm{mN/m}$ (**a-b**), 60 wt% glycerol-water mixutre with $\mu = 11.0\ \mathrm{mPa \cdot s}$ and $\gamma = 64.7\ \mathrm{mN/m}$ (**c-d**), 0.14 wt% SDS solution with $\mu = 0.9\ \mathrm{mPa \cdot s}$ and $\gamma = 35.0\ \mathrm{mN/m}$ (**e-f**) and milk with $\mu = 1.5\ \mathrm{mPa \cdot s}$ and $\gamma = 53.3\ \mathrm{mN/m}$ (**g-h**) at two impinging velocities. The red lines are the fittings of the data with the Gaussian distribution function, which gives a standard deviation (SD) of less than 15%.

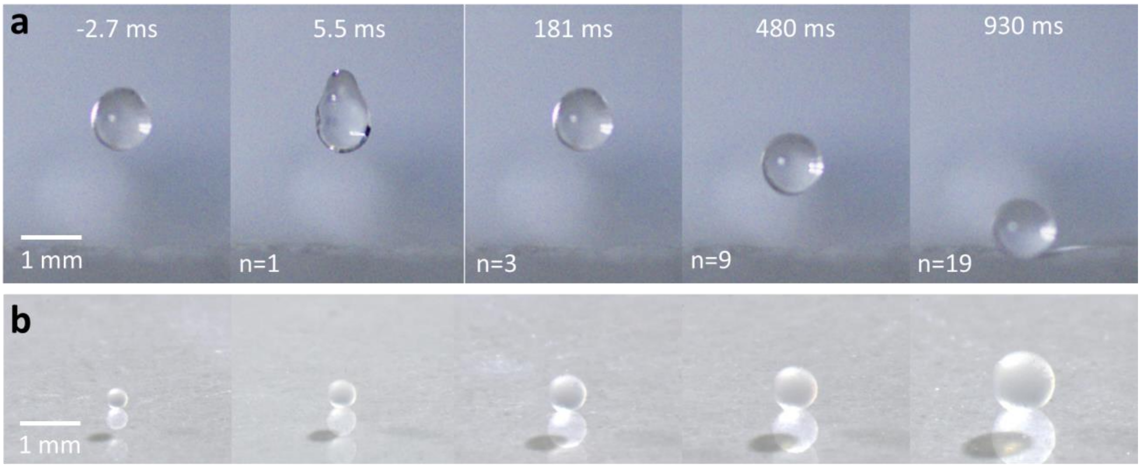

Figure S14. Fabrication of liquid marbles with 100 nm-diameter hydrophobic particles. (**a**) Snapshots of a water microdrop impinging on the hydrophobic powder, forming a powder-coated liquid marble; (**b**) liquid marbles with radii of $\sim 160\ \mu$m – $\sim 500\ \mu$m deposited on hydrophilic surfaces. It is clearly seen that liquid marbles fabricated with 100 nm-diameter hydrophobic particles are much more transparent than that fabricated with 5 μm-diameter hydrophobic particles.

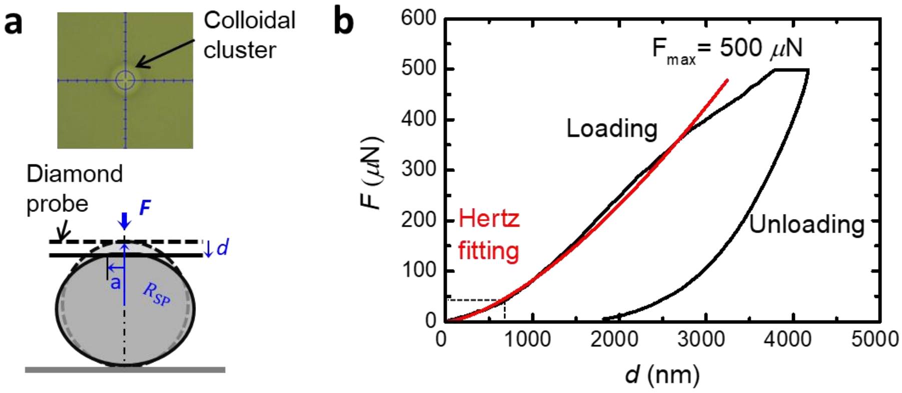

Figure S15. Mechanical characterization of supraparticles. (**a**) Top view of a supraparticle in the TI900 TriboIndenter system for precise testing location (top), and sketch of the indentation process (bottom). (**b**) A representative force-distance curve (the black line) acquired in the nanoindentation test. The red line is the fit of experimental data in the early elastic deformation regime with $d < 600$ nm and $d/R_{SP} < 3\%$ using the Hertz model (i.e. Equation (9)).

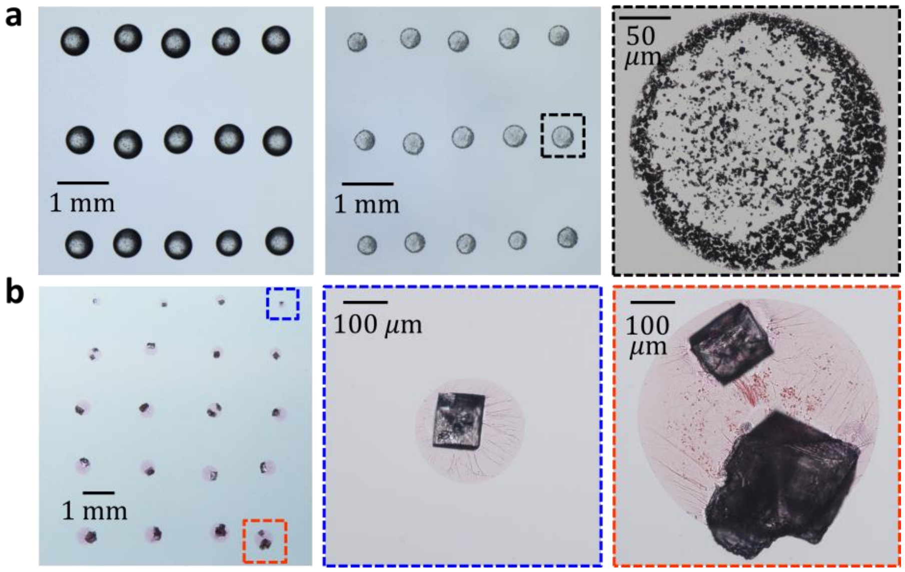

Figure S16. Patterning micromaterials using the DIμP. (**a**) Left, monodispersed droplet arrays of $0.05\ \mathrm{mg/mL}$ graphene-laden droplets with $V_d \approx 50\ \mathrm{nL}$; middle, deposited graphene patterns after evaporation; right, zoom-in view of the deposition pattern. (**b**) Left, the crystal patterns after evaporation of salty droplet arrays (from top to bottom: $V_d \approx 17\mathrm{nL}$, $80\ \mathrm{nL}$, $140\ \mathrm{nL}$, $168\mathrm{nL}$, $300\ \mathrm{nL}$, respectively) containing 3 wt% sodium chloride and dyed with red ink; middle, zoom-in view of the crystal structure denoted by the dashed blue square in the left image; right, zoom-in view of the crystal structure denoted by the dashed red square in the left image.. It is seen that the crystal size and number increase with the droplet volume.

Table S1. Comparison of the operational fluid properties and characteristics of various dispensing techniques.

Techniques	Nozzle/tip diameter [μm]	Surface tension [mN/m]	Viscosity [mPa s]	Volume	Characteristics	Literature
Dripping from nozzles	> 65	Any fluids		$>$ 1.4 μ	1. Gravity-driven dripping; 2. Non-contact dispensing; 3. Easy operation with simple operational platform; 4. Free of using sharp nozzles involving complex fabrications; 5. Good compatibility with any fluids.	Ref. [8, 25]
Ink-jet printing	$20 - 55$	$35 - 72$	$0.5 - 200$	0.01 nL - 0.1 nL	1. Piezo- or thermal-assisted drop generation; 2. Non-contact dispensing; 3. Precise operation with complex platform; 4. Special sharp nozzles needed; 5. Fail to dispense	Ref. [17-23]

					complex fluids due to clogging.	
Laser-induced jetting	$1 - 520$	$41 - 70$	$1.5 - 860$	300 fL -8 nL	1. Laser assisted jetting and drop formation; 2. Non-contact dispensing; 3. Precise operation with complex platform; 4. Special sharp capillary tubes/ultra-thin liquid film needed; 5. Compromised to dispense complex fluids due the disruption of the jet stream.	Ref. [15, 26, 27]
Dip-pen lithography	$\sim 0.001 - \sim 0.1$	Nonvolatile liquids		0.5 aL -4 fL	1. Requirement of very sharped tips; 2. Contact dispensing with the risk of tip damage; 3. Precise operation with very complex platform; 4. Nanometer-sized tips needed; 5. Fail to dispense low-surface-tension fluids, volatile fluids and complex fluids.	Ref. [11-13]
Electrohydrodynamic jetting	$0.3 - 30$	$\sim 50 - 72$	$1 - 35$	7 aL − 270 f	1. Requirement of high voltage up to 1000V; 2. Non-contact dispensing; 3. Precise operation with complex platform; 4. Sharp tips/nozzles needed; 5. Only capable of dispensing liquids with specific conductivity on special substrates.	Ref. [16, 28, 29]
Pyroelectrodynamic shooting	~ 300	−	$1.4 - 8.2$	3.6 aL − 30 nI	1. High temperature requirement (>100°C) and electric- field-assisted dispensing; 2. Non-contact dispensing but only applicable for short-distance dispensing; 3. Precise operation with very complex platform; 4. Thin liquid film/sharp tips needed; 5. Not applicable for volatile fluids.	Ref. [30]
Acoustophoretic printing	13-140	35-72	1-1000	1.2 nL − 2.1 μ	1. Acoustic force assisted dispensing; 2. Non-contact dispensing; 3. Precise operation with very complex platform; 4. Special design and fabricated nozzles needed; 5. More applicable for highly viscous fluids.	Ref. [24]
Chemically patterned surfaces	10 μm − 1 mm (pattern size)	Any fluids with different wettabilities on hydrophilic-hydrophobic patterns		0.1 nL − 1 μL	Requirement of sufficiently different adhesion force on hydrophilic-hydrophobic patterned surface; 2. Contact/Non-contact dispensing; 3. Precise operation with complex platform including dip coating, spin coating, or the control of drop/surface moving; 4. Free of using sharp nozzles involving complex fabrications but with requirement of precise design and fabrication of chemically patterned surface; 5. Good compatibility with any fluids with different wettabilities on hydrophilic-hydrophobic patterns.	Ref. [31-34]

Microfluidics	$10\ \mu$m − 1 mm (channel width)	Any liquids with requirement of one more immiscible liquid		4 pL − 1 μL	Drop generation due to hydrodynamic instabilities in microchannel; 2.-; 3. Precise operation with complex platform; 4. Microchannel with special geometries needed; 5. Good compatibility with any fluids but with requirement of one more immiscible fluid as the continuous phase.	Ref. [35-37]
Macrodrop-impact-mediated microdispenser	240	25-72	0.9 − 10	270 pL − 524 [illegible]	1. Macrodrop-impact-mediated microdispensing; 2. Non-contact dispensing; 3. Facile operation with simple, operational, portable platform; 4. Free of using sharp nozzles involving complex fabrications; 5. More applicable for fluids with low surface tensions and complex fluids.	This work

Table S2. Comparison of advantages and challenges of varisous dispensing techniques

Techniques	Advantages	Challenges	Main applications	Literature
Dripping from nozzles	Easy operation; low cost.	Low volume tunability; low droplet generation efficiency (≲ 10 Hz).	Common droplet generation.	Ref. [8, 25]
Ink-jet printing	High positioning precision (offset disdance of ~10 μm); high volume repeatability; high drop generation efficiency (up to tens of KHz).	Low volume tunability; low compatability with liquids of low surface tension; concern of clogging; high cost.	Precise displays; manufacturing; biomedical/logical applications.	Ref. [18, 38, 39]
Laser-induced jetting	High positioning precision; high volume repeatability; high droplet generation efficiency (up to tens of KHz).	Complex operation system; low capability to dispense complex	Precise displays; micromanufacturing.	Ref. [15, 26, 27]

		fluids; high cost.		
Dip-pen lithography	High positioning precision (offset disdance of ~5 nm); high volume repeatability.	Complex operation system; limited printing area; low capability to dispense volatile fluids; high cost.	Ultrahigh-density/resolution patterning; micro/nano manufacturing; genetic engineering and cell detection.	Ref. [11-13]
Electrohydrodynamic jetting	High positioning precision; high volume repeatability; high droplet generation efficiency (at KHz level).	Complex operation system; concern of clogging.	Micro displays; micromanufacturing.	Ref. [16, 28, 29]
Pyroelectrodynamic shooting	High positioning precision; high volume repeatability.	Complex operation system; limitation of printing biologically active reagent for its temperature working conditions.	Micro/nano manufacturing.	Ref. [30]
Acoustophoretic printing	High positioning precision (offset disdance of ~100 μm); high volume repeatability; high compatibility for printing viscous liquids; high droplet generation efficiency (at KHz level).	Complex operation system; low compatability with liquids of low surface tension; concern of clogging.	Precise displays; Micromanufacturing; biological applications.	Ref. [24]
Chemically patterned surfaces	High positioning precision.	Complex fabrication of hydrophilic-hydrophobic	Generation of droplet arrays; biomedical/logical applications.	Ref. [35-37]

		surface; high dependence on surface chemical patterns.		
Microfluidics	Fine control over microenvironment; high volume repeatability.	Complex operation system (specially designed microchannels, external pumps, connectors and valves); concern of clogging; high dependence on microchannel geometry; predication of drop size under different conditions.	Microreactors; microanalysis; micromanufacturing.	Ref. [40-42]
Macrodrop-impact-mediated microdispenser	Easy operation; high volume repeatability; high volume tunability; high compatability with complex liquids and liquids of low surface tension; low cost.	Low positioning precision; low compatibility with liquids of high viscosity; low droplet generation efficiency (at several Hz level).	Biological applications; micro chemical reactions; micromanufacturing.	This work

Table S3. Equilibrium (θ_{eq}), advancing (θ_a) and receding (θ_r) contact angles of 4 μL water drops measured on nine solid surfaces of different wetting properties.

Surfaces	I	II	III	IV	V	VI	VII	VIII	IX
θ_{eq} [º]	27±2	76±2	106±1	113±1	140±2	150±1	154±1	157±2	161±1
θ_a [º]	31±1	88±1	111±1	118±1	143±1	152±1	156±1	160±2	163±2
θ_r [º]	–	65±2	100±1	106±1	135±1	149±1	149±1	150±2	159±1

Table S4. Physical properties of diverse liquid mixtures studied.

Composition [weight percent wt%]			Density [kg/m^3]	Viscosity [mPa · s]	Surface tension [mN/m]	θ_{eq} [°]
Glycerol	Ethanol	Water				
0%	0%	100%	997	0.9	71.8	161±1
0%	10%	90%	982	1.3	46.6	150±2
0%	40%	60%	929	2.3	29.6	136±2
10%	10%	80%	998	1.7	46.4	143±1
10%	20%	70%	980	2.2	37.0	141±1
20%	10%	70%	1040	2.2	45.4	145±2
30%	20%	50%	1030	4.0	35.1	141±2
30%	0%	70%	1090	2.5	66.5	154±2
45%	0%	55%	1100	4.7	65.6	153±2
50%	10%	40%	1108	7.4	41.3	143±2
50%	20%	30%	1017	9.3	34.6	141±2
60%	0%	40%	1146	11.0	64.7	151±3
60%	30%	10%	1061	19.5	30.4	139±2
64%	0%	36%	1167	14.4	64.0	146±3
68%	0%	32%	1195	19.4	63.5	146±2

Composition [weight percent wt%]		Density [kg/m^3]	Viscosity [mPa · s]	Surface tension [mN/m]	θ_{eq} [°]
Ethylene Glycol	Water				
40%	60%	1064	2.2	58.3	143±1
50%	50%	1088	2.8	56.0	142±2
80%	20%	1092	6.5	50.0	142±2
100%	0%	1140	13.8	46.2	140±2

Composition [weight percent wt%]		Density [kg/m^3]	Viscosity [mPa · s]	Surface tension [mN/m]	θ_{eq} [°]
Ethylene Glycol	Ethanol				
35%	65%	860	16.7	24.3	128±2
40%	60%	867	20.3	25.1	131±1

Composition [weight percent wt%]		Density [kg/m^3]	Viscosity [mPa · s]	Surface tension [mN/m]	θ_{eq} [°]
SDS	Water				
0.029%	99.971%	997	0.9	65	149±3
0.144%	99.856%	997	0.9	35	147±3

Other liquids	Density [kg/m^3]	Viscosity [mPa · s]	Surface tension [mN/m]	θ_{eq} [°]
Dodecane	749	1.5	25.4	140±3
Cetane	773	3.1	27.1	142±2
Suspension	997	1.0	70.3	160±1
O/W emulsion	913	1.8 (at shear rate of 200 s^{-1})	32.6	140±3
Milk	1020	1.5	53.3	150±3

Supplementary movies

Movie S1. The formation of a thin jet in drop impact at $We \approx 4.1$.

Movie S2. The formation of a thick jet in drop impact at $We \approx 7.6$.

Movie S3. Drop formation from a thin jet recorded at 1 $\mu\text{m/pixel}$ and 340000 fps.

Movie S4. Drop formation from a thick jet recorded at 1 $\mu\text{m/pixel}$ and 340000 fps.

Movie S5. Demonstration of the concept of macrodrop-impact-mediated fluid microdispensing.

Movie S6. Dispensing a 34 nL aqueous iron(III) solution into a 4 μL aqueous sulfosalicylic acid drop.

Movie S7. Dispensing six aqueous iron(III) drops into a 4 μL aqueous sulfosalicylic acid drop.

Movie S8. Manipulating different chemical reactions by successive microdispensing.

Movie S9. Formation of liquid marbles by impinging microdrops in powder.

REFERENCES

[1] J.C. Burton, R. Waldrep, P. Taborek, *Phys. Rev. Lett.* **2005**, 94, 184502.
[2] M.S. Longuethiggins, B.R. Kerman, K. Lunde, *J. Fluid Mech.* **1991**, 230, 365.
[3] A.H. Woodcock, C.F. Kientzler, A.B. Arons, D.C. Blanchard, *Nature* **1953**, 172, 1144.
[4] J.S. Lee, B.M. Weon, S.J. Park, J.H. Je, K. Fezzaa, W.K. Lee, *Nat. Commun.* **2011**, 2, 367.
[5] H.Y. Erbil, *Adv. Colloid Interface Sci.* **2012**, 170, 67.
[6] A.C. Best, *Q. J. R. Meteorol. Soc.* **1952**, 78, 200.
[7] J.C. LASHERAS, *J. Fluid Mech.* **1999**, 383, 307.
[8] T. Tate, *Phil. Mag.* **1864**, 27, 176.
[9] H. Hertz, *MacMillan: London* **1896**, 163.
[10] H. Ghaednia, X. Wang, S. Saha, Y. Xu, A. Sharma, R. Jackson, *Appl. Mech. Rev.* **2017**, 69, 060804.
[11] Lee, K.-B., *Science (New York, N.Y.)* **2002**, 295, 1702.
[12] D.S. Ginger, H. Zhang, C.A. Mirkin, *Angew. Chem. Int. Ed.* **2004**, 43, 30.
[13] K. Salaita, Y. Wang, C.A. Mirkin, *Nature Nanotech.* **2007**, 2, 145.
[14] W. Shim, A.B. Braunschweig, X. Liao, J.N. Chai, J.K. Lim, G.F. Zheng, C.A. Mirkin, *Nature* **2011**, 469, 516.
[15] Y. Tagawa, N. Oudalov, C.W. Visser, I.R. Peters, D. van der Meer, C. Sun, A. Prosperetti, D. Lohse, *Phys. Rev. X* **2012**, 2, 1.
[16] J.-U. Park, M. Hardy, S. Kang, K. Barton, K. Adair, D. Mukhopadhyay, M. Strano, A.G. Alleyne, J. Georgiadis, P. Ferreira, J. Rogers, *Nature Mater.* **2007**, 6, 782.
[17] T. Shimoda, S. Kanbe, H. Kobayashi, S. Seki, C.R. Towns, *SID Int. Symp. Digest Tech. Papers* **1999**, 30, 376.
[18] B. Derby, *Annu. Rev. Mater. Res.* **2010**, 40, 395.
[19] P. Calvert, *Chem. Mater.* **2001**, 13, 3299.
[20] B.-J. de Gans, P.C. Duineveld, U.S. Schubert, *Adv. Mater.* **2004**, 16, 203.
[21] M.M. Mohebi, J.R.G. Evans, *J. Comb. Chem.* **2002**, 4, 267.
[22] R.E. Saunders, J.E. Gough, B. Derby, *Biomaterials* **2008**, 29, 193.
[23] C.D. Paul, M.d.K. Margreet, B. Michael, S. Aad, A.H.M. Kees, W. Peter van de, G.J.C. Ivo, B. Ton van de, J.M.R. Jan-Eric, I.H. Eliav, *Proc. SPIE* **2002**, 4464, 59.
[24] D. Foresti, K.T. Kroll, R. Amissah, F. Sillani, K.A. Homan, D. Poulikakos, J.A. Lewis, *Sci. Adv.* **2018**, 4, eaat1659.
[25] Pei-Hsun, Tsai, An-Bang, Wang, *Langmuir* **2019**, 35, 4763.
[26] M.S. Brown, C.F. Brasz, Y. Ventikos, C.B. Arnold, *J. Fluid Mech.* **2012**, 709, 341.
[27] Y. Lin, Y. Huang, D. Chrisey, *J. Appl. Phys.* **2009**, 105, 093111.
[28] J. Fernandez de la Mora, *Annu. Rev. Fluid Mech.* **2007**, 39, 217.
[29] T. Achtzehn, R. Müller, D. Duft, T. Leisner, *Eur. Phys. J. D* **2005**, 34, 311.
[30] P. Ferraro, S. Coppola, S. Grilli, M. Paturzo, V. Vespini, *Nature Nanotech.* **2010**, 5, 429.
[31] Y. Huang, F. Li, M. Qin, L. Jiang, Y. Song, *Angew. Chem. Int. Ed.* **2013**, 52,
[32] Y. Lin, Z. Wu, Y. Gao, J. Wu, W. Wen, *Appl. Surf. Sci.* **2018**, 442, 189.
[33] K.W. Yong, P.B. Ganesan, M.S.N. Kazi, S. Ramesh, I.A. Badruddin, N.M. Mubarak, *Phys. Fluids* **2018**, 30, 122006.
[34] H. Li, A. Li, Z. Zhao, M. Li, Y. Song, *Small Struct.* **2020**, 1, 2000028.
[35] N. Tarchichi, F. Chollet, J.-F. Manceau, *Microfluid. Nanofluid.* **2013**, 14, 45.
[36] P. Zhu, L. Wang, *Lab Chip* **2017**, 17, 34.
[37] S.-Y. Teh, R. Lin, L.-H. Hung, A.P. Lee, *Lab Chip* **2008**, 8, 198.
[38] G. Cummins, M.P.Y. Desmulliez, *Circuit World* **2012**, 38, 193.
[39] K.-S. Kwon, M.K. Rahman, T.H. Phung, S. Hoath, S. Jeong, J.S. Kim, *Flexible Printed Electron.* **2020**, 043003.
[40] E. Dressaire, A. Sauret, *Soft Matter* **2017**, 13, 37.

[41] G.M. Whitesides, *Nature* **2006**, 442, 368.
[42] H. Gu, M.H. Duits, F. Mugele, *Int. J. Mol. Sci.* **2011**, 12, 2572.
